# Comparative Assessment of Severe Acute Respiratory Syndrome Coronavirus 2 Variants in the Ferret Model

**DOI:** 10.1128/mbio.02421-22

**Published:** 2022-09-22

**Authors:** Joanna A. Pulit-Penaloza, Jessica A. Belser, Xiangjie Sun, Claudia Pappas, Nicole Brock, Troy J. Kieran, Jana M. Ritter, Josilene N. Seixas, Joyce Jones, Poulami Basu Thakur, Elizabeth Pusch, Li Wang, Terrence M. Tumpey, David E. Wentworth, Bin Zhou, Taronna R. Maines

**Affiliations:** a Influenza Division, National Center for Immunization and Respiratory Diseases, Centers for Disease Control and Prevention, Atlanta, Georgia, USA; b Division of High-Consequence Pathogens and Pathology, National Center for Emerging and Zoonotic Infectious Diseases, Centers for Disease Control and Prevention, Atlanta, Georgia, USA; Johns Hopkins Bloomberg School of Public Health

**Keywords:** SARS-CoV-2, ferret, reinfection, airborne transmission, variant, Delta, COVID-19

## Abstract

The continued spread of severe acute respiratory syndrome coronavirus 2 (SARS-CoV-2) in humans necessitates evaluation of variants for enhanced virulence and transmission. We used the ferret model to perform a comparative analysis of four SARS-CoV-2 strains, including an early pandemic isolate from the United States (WA1), and representatives of the Alpha, Beta, and Delta lineages. While Beta virus was not capable of pronounced replication in ferrets, WA1, Alpha, and Delta viruses productively replicated in the ferret upper respiratory tract, despite causing only mild disease with no overt histopathological changes. Strain-specific transmissibility was observed; WA1 and Delta viruses transmitted in a direct contact setting, whereas Delta virus was also capable of limited airborne transmission. Viral RNA was shed in exhaled air particles from all inoculated animals but was highest for Delta virus. Prior infection with SARS-CoV-2 offered varied protection against reinfection with either homologous or heterologous variants. Notable genomic variants in the spike protein were most frequently detected following WA1 and Delta virus infection.

## INTRODUCTION

Since its first detection in December 2019, severe acute respiratory syndrome coronavirus 2 (SARS-CoV-2), the etiologic agent of coronavirus disease 2019 (COVID-19), has caused the most profound pandemic of modern times, resulting in millions of deaths worldwide (1). This enveloped, positive-sense, single-stranded RNA virus of the *Coronaviridae* family causes zoonotic disease (2) but can also infect humans via binding of the virus spike (S) glycoprotein to the angiotensin-converting enzyme 2 (ACE2) receptor of the human respiratory epithelium (3). Sustained worldwide transmission of SARS-CoV-2 has rapidly given rise to established lineages in various geographical locations. Notable variants include B.1.1.7 (Alpha), B.1.351 (Beta), P.1 (Gamma), B.1.617.2 (Delta), and B.1.1.529 (Omicron) (4). As variants have emerged and SARS-CoV-2 has continued to adapt to humans, in some cases, enhanced transmission and virulence have been observed.

A critical component of understanding the pandemic has been establishment of models for SARS-CoV-2 pathogenesis and transmission. Unfortunately, no one mammalian model fully recapitulates both the disease features of COVID-19 and the transmissibility of SARS-CoV-2 (5). The ferret is a well-established animal model for respiratory pathogens, especially influenza viruses (6), and allows evaluation of SARS-CoV-2 transmission using a permissive direct contact transmission (DCT) model (inclusive of direct and indirect contact and airborne routes between cohoused animals) or a respiratory droplet transmission (RDT) model (restricted to transmission mediated via droplets and fine aerosol particles) (7, 8). Furthermore, quantification and size fractionation of virus-laden aerosols emitted from infected ferrets are possible, providing enhanced understanding of this dynamic process (9). Widespread emergence of antigenically distinct SARS-CoV-2 variants has led to both primary human infection and reinfection of previously infected humans (10). However, considering the broad immunological landscape of the human population (especially after licensure and administration of SARS-CoV-2 vaccines) and the diversity of exposure routes and doses possible to establish infection, assessments of the basic reproduction number (R_0_) at the population level do not fully capture the intrinsic and relative capacity for transmission of newly emerged SARS-CoV-2 variants (11). Here, we established DCT and RDT models with an original SARS-CoV-2 isolate (USA-WA1/2020 [WA1]) as a baseline in ferrets and compared the pathogenicity and transmissibility of this reference virus during primary infection to those of a panel of Alpha, Beta, and Delta variant viruses. We then evaluated protection from reinfection and studied how both primary and reinfection scenarios modulated virus shedding kinetics (in nasal washes [NW] and in exhaled aerosols), pathology, and emergence of genetically variant populations. We found that viral RNA of all SARS-CoV-2 strains studied could be detected in exhaled aerosols from inoculated ferrets and that prior infection affected reinfection kinetics in a strain-specific manner. These studies demonstrate the utility of the ferret model to evaluate SARS-CoV-2 infection and transmission and offer a greater understanding of how prior infection can modulate subsequent infection with a heterologous SARS-CoV-2, contributing to our ability to assess new and emerging variants.

## RESULTS

### SARS-CoV-2 disease, dissemination, and pathology in ferrets.

Four SARS-CoV-2 strains (WA1, Alpha, Beta, and Delta), each representing a different lineage and displaying key amino acid differences in the S protein (see Table S1 in the supplemental material), were evaluated for pathogenicity and transmissibility using a ferret model. Following intranasal inoculation, mild disease was observed in ferrets over a 2-week observation period, with no overt respiratory signs of infection, such as nasal discharge or sneezing. Lethargy was noted only in Delta virus-infected ferrets, where it was mild and lasted for 1 to 2 days. Weight loss was not consistently observed among animals for any of the virus groups but was highest for the Alpha and Delta virus groups (Table 1). Fever was more frequently detected than weight loss but was modest and transient; fever reached the highest levels for the WA1 and Delta viruses.

**TABLE 1:** Comparison of clinical signs and virus shedding in SARS-CoV-2-infected ferrets

Challenge virus	Wt loss (%)^*a*^	Temp increase (C°)^*b*^	NW titer (log_10_ PFU/mL)^*c*^	NW titer (log_10_ copies/mL)^*d*^	RS titer (log_10_ copies/mL)^*d*^	CW titer (log_10_ copies/mL)^*d*^	Aerosol titer (log_10_ copies/L)^*e*^
Primary challenge							
WA1	0.9 (3/6)	1.3 (5/6)	4.5 (6/6, days 3–5)	8.4 (6/6, days 3–7)	7.2 (6/6, days 3–5)	4.3 (5/6, days 3–7)	3.0 (3/3, days 3–7)
Alpha	3.0 (3/6)	0.9 (5/6)	4.4 (6/6, days 1–3)	8.1 (6/6, days 1–3)	6.5 (6/6, days 1–7)	3.5 (3/6, days 3–5)	3.7 (3/3, day 2)
Beta	0.0 (0/6)	0.6 (6/6)	1.4 (1/6, day 1)	4.5 (6/6, day 1)	3.8 (1/6, day 15)	<2.9	1.7 (3/3, days 1–2)
Delta	3.3 (4/6)	1.7 (6/6)	5.1 (6/6, day 1)	9.6 (6/6, day 3)	6.0 (6/6, days 1–11)	4.5 (6/6, days 3–7)	4.2 (3/3, day 2)
Primary/Secondarychallenge							
WA1							
WA1	2.6 (2/2)	0.1 (1/2)	<1	3.8 (2/2, day 1)	2.5 (1/2, day 5)	NT^*f*^	2.1 (2/2, days 2 and 8)
Alpha	1.4 (2/2)	0.6 (1/2)	3.1 (2/2, day 1)	5.3 (2/2, day 1)	3.1 (2/2, day 1)	NT	2.0 (2/2, days 1 and 6)
Beta	0.3 (1/2)	0.8 (2/2)	<1	3.6 (2/2, day 1)	<2.9	NT	0.9 (1/2, day 4)
Delta	2.1 (1/2)	0.4 (1/2)	2.7 (2/2, day 1)	5.0 (2/2, day 1)	<2.9	NT	1.1 (2/2, days 1 and 2)
Alpha							
WA1	7.8 (2/2)	1.1 (2/2)	<1	4.6 (2/2, day 1)	<2.9	NT	1.4 (2/2, days 1 and 2)
Alpha	7.9 (2/2)	1.8 (2/2)	<1	5.9 (2/2, day 1)	<2.9	NT	<1
Beta	NT	NT	NT	NT	NT	NT	NT
Delta	7.0 (2/2)	1.1 (2/2)	2.2 (2/2, day 1)	6.4 (2/2, days 1 and 3)	4.3 (1/2, day 5)	NT	1.2 (1/2, day 2)
Beta							
WA1	5.9 (2/2)	0.8 (2/2)	4.7 (2/2, days 3 and 7)	7.7 (2/2, days 5 and 7)	<2.9	NT	3.7 (2/2, day 7)
Alpha	4.6 (2/2)	0.8 (2/2)	4.2 (2/2, days 1 and 3)	8.6 (2/2, days 1 and 3)	5.9 (2/2, days 1 and 3)	NT	3.1 (2/2, days 4 and 6)
Beta	3.1 (2/2)	0.4 (2/2)	1.5 (2/2, days 5 and 7)	6.1 (2/2, days 3 and 7)	<2.9	NT	1.8 (2/2, day 6)
Delta	NT	NT	NT	NT	NT	NT	NT
Delta							
WA1	4.7 (2/2)	0.9 (2/2)	1.0 (1/2, day 1)	3.8 (2/2, day 1)	5.4 (2/2, day 11)	NT	1.9 (2/2, day 1)
Alpha	5.5 (2/2)	1.4 (2/2)	2.6 (2/2, day 1)	6.0 (2/2, days 1 and 3)	3.4 (1/2, day 3)	NT	2.6 (2/2, days 1 and 2)
Beta	3.0 (2/2)	1.4 (2/2)	<1	4.6 (1/2, day 1)	<2.9	NT	2.8 (2/2, day 1)
Delta	4.5 (2/2)	0.7 (2/2)	2.9 (2/2, days 1 and 3)	6.0 (2/2, days 1 and 3)	<2.9	NT	2.8 (2/2, day 2)

a Average percent maximum weight loss within 15 days postinoculation. Number of ferrets displaying weight loss over the total number of animals is in parentheses.

b Average maximum temperature change (C°) relative to baseline within 15 days postinoculation. Number of ferrets displaying temperature change over the total number of animals is in parentheses.

c Average maximum infectious virus titer in nasal wash (NW). Number of ferrets with detectable virus over the total number of animals and peak day(s) are shown in parentheses. Infectious virus titers in rectal swab (RS) and conjunctival wash (CW) samples were below the level of detection of 1 log_10_ PFU/mL.

d Average maximum viral RNA titer. Number of ferrets with detectable RNA over the total number of animals and peak day(s) are shown in parentheses. Limit of detection, 2.9 log_10_ copies/mL.

e Average maximum viral RNA titer in aerosol samples; peak day(s) in parentheses. Limit of detection, 1 log_10_ copy/L of air.

f NT, not tested.

10.1128/mbio.02421-22.2 TABLE S1 SARS-CoV-2 strains used in the study. SARS-CoV-2 strains used in the study; lineage, passage history, GISAID accession number, and amino acid mutations in the S protein are listed. Download Table S1, PDF file, 0.01 MB. Copyright © 2022 Pulit-Penaloza et al. 2022 Pulit-Penaloza et al. https://creativecommons.org/licenses/by/4.0/ This content is distributed under the terms of the Creative Commons Attribution 4.0 International license.

**FIG 1:**
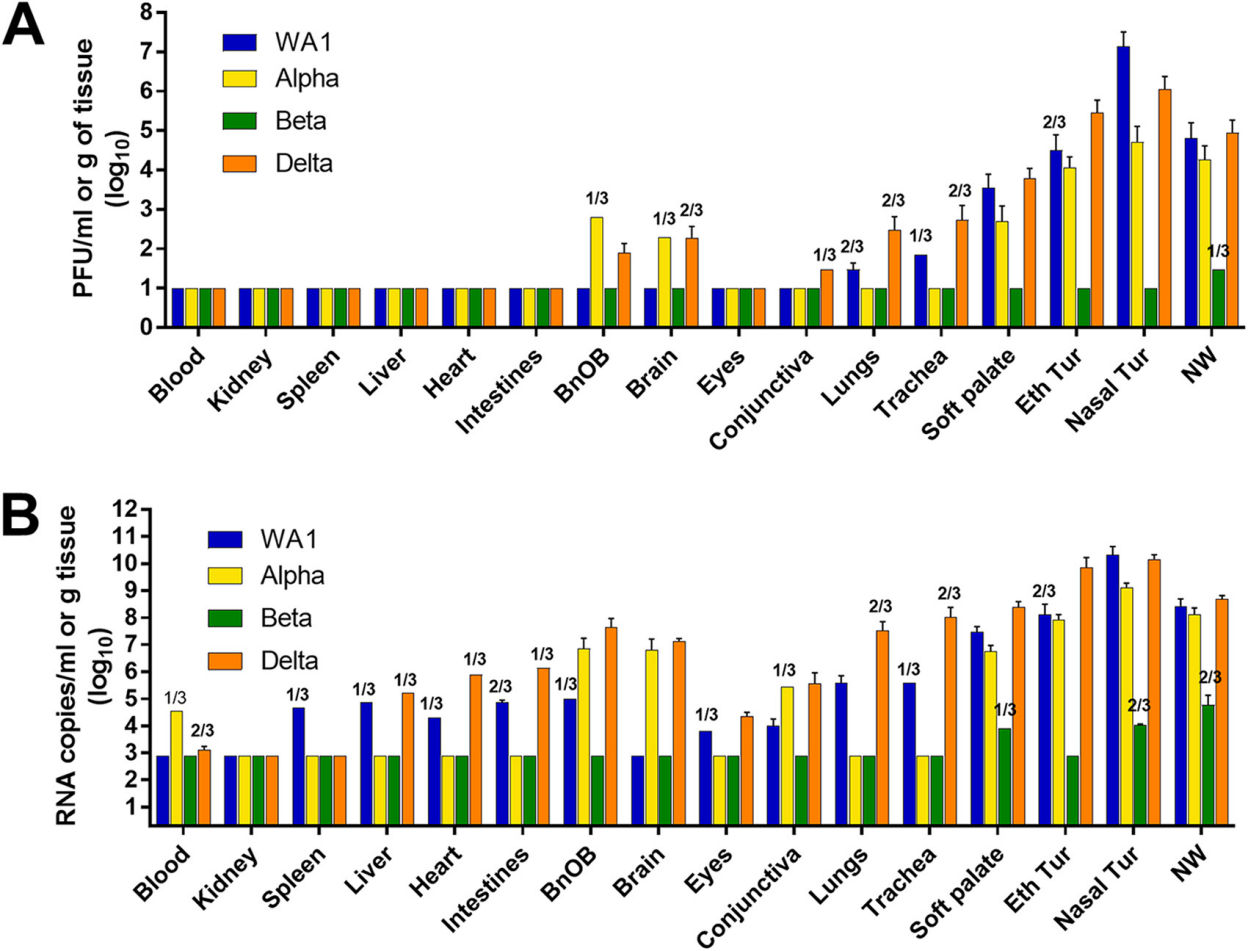
SARS-CoV-2 dissemination in ferret tissues. Ferrets were inoculated with 6.0 log_10_ PFU of WA1, Alpha, Beta, or Delta virus, and tissues were collected on day 3 p.i. Virus titers were evaluated using standard plaque assay to determine infectious virus load (limit of detection, 1.0 log_10_ PFU/mL or g) (A) and real-time quantitative RT-PCR (qRT-PCR) to determine viral RNA load (limit of detection, 2.9 log_10_ RNA copies/mL or g) (B). Mean titers and standard deviation were calculated for each sample type; titers are reflective of *n* = 3 samples with detectable virus unless indicated. Titers in blood, eyes, conjunctiva, soft palate, ethmoid turbinate (Eth Tur), nasal turbinate (Nasal Tur), and NW are presented as log_10_ PFU or RNA copies/milliliter, and titers in kidney, spleen, liver, heart, intestines (pooled duodenum, jejuno-ileal loop, and descending colon), olfactory bulb (BnOB), brain (pooled anterior and posterior brain), lungs, and trachea are presented as log_10_ PFU or RNA copies/gram of tissue.

Virus dissemination throughout and beyond the respiratory tract was examined in specimens collected on day 3 postinoculation (p.i.) from 16 sites of 3 inoculated ferrets for each virus group. Infectious virus was predominately limited to the respiratory tract, with few exceptions; notably, Alpha and Delta variants were detected in olfactory bulb and brain tissues (Fig. 1A). WA1, Alpha, and Delta viruses were at highest titers in the nasal turbinate tissues (7.1, 4.7, and 6.1 log_10_ PFU/mL, respectively), while infectious Beta virus was recovered at a low titer in one NW specimen only. Mean peak infectious virus titers and frequencies of detection gradually fell with each tissue that was collected deeper into the respiratory tract. Only WA1 and Delta viruses were detected in lungs and at a low titer (≤2.5 log_10_ PFU/g). SARS-CoV-2 RNA was much more prevalent in tissues than was infectious virus, and up to 3 to 4 orders of magnitude higher, depending on the strain and tissue (Fig. 1B). In agreement with infectious titers, WA1 and Delta viral RNA were at the highest levels in the upper respiratory tract tissues compared with the other variants; Beta virus RNA was detected only in soft palate, nasal turbinate, and NW specimens, demonstrating that this variant displayed the least pathogenic potential of all the viruses tested in the ferret model.

Concurrent with viral quantification, histopathological examination of trachea, lung, and extrapulmonary tissues collected from three ferrets per virus on day 3 p.i. was performed. No significant differences were observed among virus groups or between virus-inoculated and naive ferrets (Fig. S1). Trachea samples showed diffusely intact, ciliated respiratory epithelium, and either no or very rare scattered lymphocytic infiltrates within the subepithelial stroma were noted. Lung tissues showed variable congestion, but no virus-attributable pathology was seen in any animal. Bronchi and bronchioles were free of degenerative or inflammatory changes, and no significant interstitial or intra-alveolar inflammatory infiltrates were seen. Interstitial infiltrates were absent to minimal and did not differ among groups. Diffuse alveolar damage was not seen in any animal. Extrapulmonary tissues had no significant histopathologic changes (data not shown). Collectively, these findings show that young ferrets (≤8 months old) do not represent a model for severe COVID-19 pneumonia but rather display a short-lived, mild respiratory disease upon SARS-CoV-2 infection with no virus-attributable pathology.

10.1128/mbio.02421-22.1 FIG S1 Histopathology of the respiratory tract of SARS-CoV-2-inoculated ferrets. Trachea samples (top row) from naive and SARS-CoV-2-infected animals show intact, ciliated respiratory epithelium and no significant submucosal inflammation. Lung samples (bottom row) from all animals show bronchioles (*) free of degenerative or inflammatory changes, alveolar spaces are collapsed (tissue collection artifact), and alveolar walls are diffusely congested. Download FIG S1, PDF file, 0.3 MB. Copyright © 2022 Pulit-Penaloza et al. 2022 Pulit-Penaloza et al. https://creativecommons.org/licenses/by/4.0/ This content is distributed under the terms of the Creative Commons Attribution 4.0 International license.

### SARS-CoV-2 shedding and transmission in ferrets.

**FIG 2:**
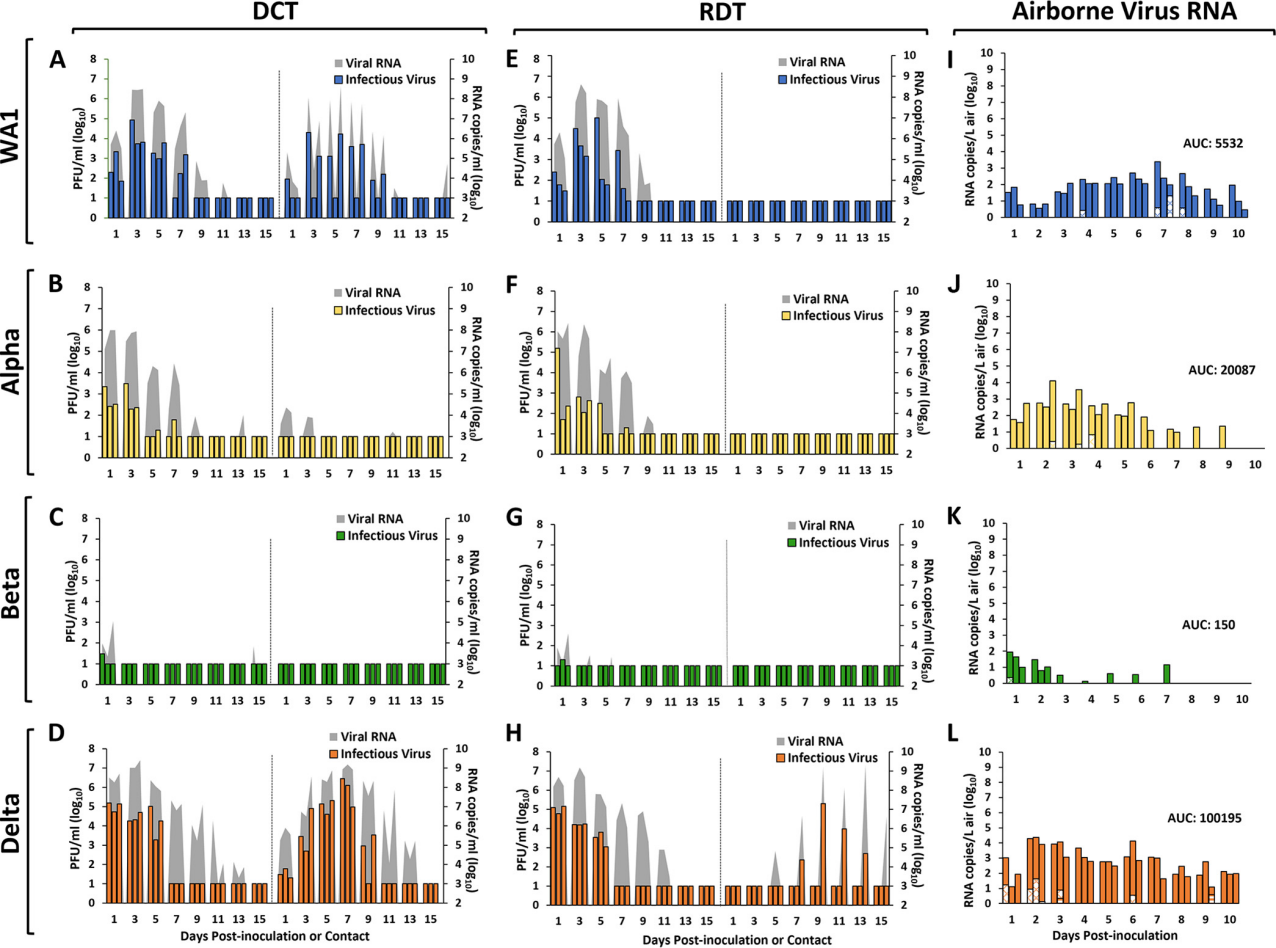
SARS-CoV-2 transmission and airborne viral RNA collected from inoculated ferrets. Ferrets were inoculated with 6.0 log_10_ PFU of SARS-CoV-2: WA1 (A and E), Alpha (B and F), Beta (C and G), or Delta (D and H). After 24 h, transmission was assessed either by adding a naive ferret to each cage housing an inoculated ferret (direct contact transmission [DCT] model) or by placing a naive ferret in an adjacent cage (respiratory droplet transmission [RDT] model) (3 ferret pairs/virus). Virus titers in NW samples collected from individual inoculated or contact ferrets are shown on the left or right side of each panel, respectively. Virus titers were evaluated using standard plaque assay to determine infectious virus load (left *y* axis, limit of detection = 1.0 log_10_ PFU/mL) and real-time quantitative RT-PCR to determine viral RNA load (right *y* axis, limit of detection = 2.9 log_10_ RNA copies/mL). Aerosol samples were collected from each inoculated animal included in the RDT experiments (*n* = 3 per virus) every day for 10 days p.i. Air samples from ferrets inoculated with WA1 (I), Alpha (J), Beta (K), and Delta (L) were collected from each ferret for 1 h using a cyclone sampler which separated aerosol particles based on size into three fractions: >4 μm (solid bars), 1 to 4 μm (dotted bars), and <1 μm (striped bars). Viral RNA in each sample was quantified using real-time qRT-PCR, and the data for each individual ferret and each aerosol fraction are shown in the form of a stacked bar. Limit of detection = 1 RNA copy/L of air.

Groups of six ferrets each were intranasally inoculated with SARS-CoV-2, and every 2 days NW, rectal swab (RS), and conjunctival wash (CW) samples were collected. Ferrets inoculated with the Delta variant had the highest NW titers on day 1 p.i. at 5.1 log_10_ PFU/mL. Ferrets inoculated with WA1 or Alpha virus reached mean peak NW titers of 4.4 to 4.5 log_10_ PFU/mL, with peak titers of WA1 virus delayed relative to those of Delta or Alpha viruses (Table 1; Fig. 2). In contrast, infectious Beta variant was detected only on day 1 p.i. (mean peak titers of 1.4 log_10_ PFU/mL), but none of the animals seroconverted (Table S2), indicating the Beta viral RNA detection was attributed to residual inoculum. Infectious virus was not recovered in any RS or CW collected in this study. In contrast, viral RNA was detected consistently and at a high titer in RS specimens from ferrets inoculated with WA1, Alpha, and Delta viruses; CW specimens were also positive for viral RNA, albeit at reduced frequency and titer. In each case, detection of RNA in RS or CW specimens coincided with the detection of viral RNA in NW samples collected on the same day.

10.1128/mbio.02421-22.3 TABLE S2 Analysis of ferret serum collected during SARS-CoV-2 transmission experiments. ^a^All ELISAs were performed using recombinant SARS-CoV-2 S1+S2 proteins. OD_450_ values for IgG were normalized by subtracting the ELISA cutoff value (negative-control mean OD [0.06 to 0.44] + 3× STDEV [standard deviation]). Each cell shows data for an individual ferret. Negative, below the ELISA cutoff value. ^b^Direct contact transmission model (DCT); respiratory droplet transmission model (RDT). ^c^Ferrets were inoculated with 6.0 log_10_ PFU of SARS-CoV-2, and sera were collected on day 19 or 20 postinoculation (p.i.) or postcontact (p.c.). Download Table S2, PDF file, 0.01 MB. Copyright © 2022 Pulit-Penaloza et al. 2022 Pulit-Penaloza et al. https://creativecommons.org/licenses/by/4.0/ This content is distributed under the terms of the Creative Commons Attribution 4.0 International license.

The same six inoculated ferrets for each virus group were also used as donors in DCT and RDT experiments to evaluate the transmission capabilities of the SARS-CoV-2 strains. In the DCT experiment, WA1 virus-inoculated ferrets shed infectious virus until day 7 p.i. with the highest titers on day 3 p.i. (Fig. 2A), and viral RNA was detected in NW samples from all three contact ferrets, though only two shed infectious virus and seroconverted by the end of the study (Table S2). Ferrets inoculated with Alpha virus displayed mean peak titers early after inoculation with viral clearance by days 3 to 7 p.i. (Fig. 2B; Table S2). Although respective contact ferrets had viral RNA detected in NW specimens, no infectious virus was recovered, and none of the animals seroconverted to the challenge virus. As mentioned above, Beta virus was not capable of pronounced replication in ferrets; none of the contact animals had detectable virus in NW, and seroconversion was not observed (Fig. 2C; Table S2). Delta virus replicated to the highest titers in the inoculated animals, and transmission was evident in 3/3 contact animals within the first day of cohousing (Fig. 2D). Infectious virus shedding peaked in the contacts on day 7 postcontact (p.c.) (6.1 log_10_ PFU/mL) and was an order of magnitude higher than in the inoculated group, and virus shedding persisted for 7 to 9 days p.c.; all contacts seroconverted to the challenge virus (Table S2). None of the contact animals shed infectious virus in RS samples at any time point for any of the SARS-CoV-2 strains; however, viral RNA was detected in RS collected from the WA1 and Delta contact ferrets that also shed infectious virus in NW, in addition to 1/3 contacts in the Alpha virus group (Table 1).

Among the four viruses examined using the RDT model (Fig. 2E to H), the Delta variant was the only virus capable of airborne transmission; 1/3 contact ferrets shed infectious virus that peaked at 5.3 log_10_ PFU/mL on day 9 p.i., and viral RNA was detected in the NW of 2/3 contacts. Seroconversion was detected only for the RD contact animal that had detectable infectious virus present in NW samples (Table S2), demonstrating that viral RNA detection alone does not confirm successful transmission. The single Delta virus contact ferret also had viral RNA present in RS samples. No other contact ferret in any of the RDT experiments had detectable virus (infectious or RNA) in NW samples.

To determine whether transmission phenotypes are reflected by the relative levels of exhaled SARS-CoV-2, air sampling was performed daily for 10 days using the three inoculated ferrets from each virus group that were donor ferrets in the RDT experiments. Sampling was performed using National Institute for Occupational Safety and Health (NIOSH) cyclone samplers that separate airborne particles into three fractions: >4 μm, 1 to 4 μm, and <1 μm. The kinetics of virus RNA shedding into the air varied by virus (Table 1; Fig. 2I to L), but Delta virus displayed the highest overall airborne shedding (mean peak of 4.2 log_10_ RNA copies/L of air). Values for area under the curve (Delta, 100,195; Alpha, 20,087; WA1, 5,532; Beta, 150) for time courses (inclusive of all fractions and ferrets combined) revealed that overall airborne Delta virus RNA was at least 5 times higher than that of any of the other viruses. Ferrets inoculated with WA1 and Delta viruses shed virus for the full 10-day time course, while Alpha and Beta viruses were exhaled from all animals for 5 and 2 days p.i., respectively (Fig. 2I to L). Although most viral RNA was detected in the >4-μm fraction, SARS-CoV-2 RNA was sporadically detected in the 1- to 4-μm fraction for all viruses. Furthermore, Delta virus RNA was the only virus detected in the <1-μm fraction (2/3 animals on days 3 and 9 p.i.). Over the course of an hour, at the peak of shedding, an average of 6.6 log_10_ Delta RNA copies were exhaled into the air by infected ferrets. WA1, Alpha, and Beta viruses were shed at 5.3, 6.0, and 4.0 log_10_ RNA copies per hour, respectively.

### Reinfection of recovered ferrets by SARS-CoV-2.

Enzyme-linked immunosorbent assay (ELISA) of sera collected 83 days following primary challenge showed that ferrets initially inoculated with WA1, Alpha, or Delta virus had IgG antibodies against homologous and heterologous SARS-CoV-2 (Table 2), suggesting that these ferrets may display some level of protection from subsequent rechallenge. In contrast, ferrets inoculated with Beta virus as a primary challenge had no detectable IgG antibodies against the S protein from WA1, Alpha, and Beta viruses (Delta was not tested). To determine whether infection with SARS-CoV-2 affected subsequent infection by homologous or heterologous virus, ferrets that were infected (via inoculation or in a direct contact setting) in the primary challenge round of experiments were rechallenged with each secondary challenge virus (*n* = 2 per virus). Clinical signs observed during the secondary infections were mild with no respiratory symptoms or lethargy noted. Fever did not exceed 1.8°C above baseline, on par with primary infection (Table 1). Although weight loss was somewhat enhanced in nearly all animals after rechallenge, it remained moderate with a maximum mean loss of up to 7.9% of baseline body weight for any of the virus groups.

**TABLE 2:** ELISA of ferret serum collected following primary and secondary challenge with homologous or heterologous SARS-CoV-2

Primary challenge virus	Secondary challenge virus^*a*^											
	WA1			Alpha			Beta			Delta		
	Days 19–20^*b*^	Day 83 (day −1)^*c*^	Day 112 (day 28)^*d*^	Days 19–20^*b*^	Day 83 (day −1)^*c*^	Day 112 (day 28)^*d*^	Days 19–20^*b*^	Day 83 (day −1)^*c*^	Day 112 (day 28)^*d*^	Days 19–20^*b*^	Day 83 (day −1)^*c*^	Day 112 (day 28)^*d*^
WA1	1.30	1.97	2.09	NT	0.77	1.50	NT	1.56	1.62	NT	1.54	1.93
	0.61	1.12	1.36	NT	0.53	2.47	NT	0.84	0.75	NT	1.53	1.83

Alpha	NT	1.21	1.67	2.04	1.47	2.49	NT	NT	NT	NT	1.47	1.76
	NT	0.67	0.78	2.09	1.88	2.09	NT	NT	NT	NT	1.42	1.67

Beta	NT	Negative	1.55	NT	Negative	1.94	Negative	Negative	1.18	NT	NT	NT
	NT	Negative	1.57	NT	Negative	1.61	Negative	Negative	0.86	NT	NT	NT

Delta	NT	1.47	1.66	NT	1.82	1.93	NT	1.54	1.35	1.56	1.74	1.74
	NT	1.36	2.06	NT	1.92	2.27	NT	1.66	1.87	1.51	1.50	1.74

a Ferrets were rechallenged (*n* = 2) with homologous or heterologous SARS-COV-2 strains 12 weeks (day 84 postinoculation) following primary challenge. All ELISAs were performed using recombinant SARS-CoV-2 S1+S2 proteins. OD_450_ values for IgG were normalized by subtracting the ELISA cutoff value (negative-control mean OD [0.06 to 0.44] + 3× STDEV [standard deviation]). Each cell shows data for an individual ferret. NT, not tested. Negative, below the ELISA cutoff value. Shading reflects results following homologous virus rechallenge.

b Sera were collected from ferrets 19 to 20 days after primary inoculation and tested by ELISA using homologous virus recombinant S proteins.

c Sera were collected 83 days after primary challenge (1 day prior to secondary challenge) and were tested using recombinant S proteins of the rechallenge virus.

d Sera were collected 112 days after primary challenge (28 days after secondary challenge) and were tested using recombinant S proteins of the rechallenge virus.

**FIG 3:**
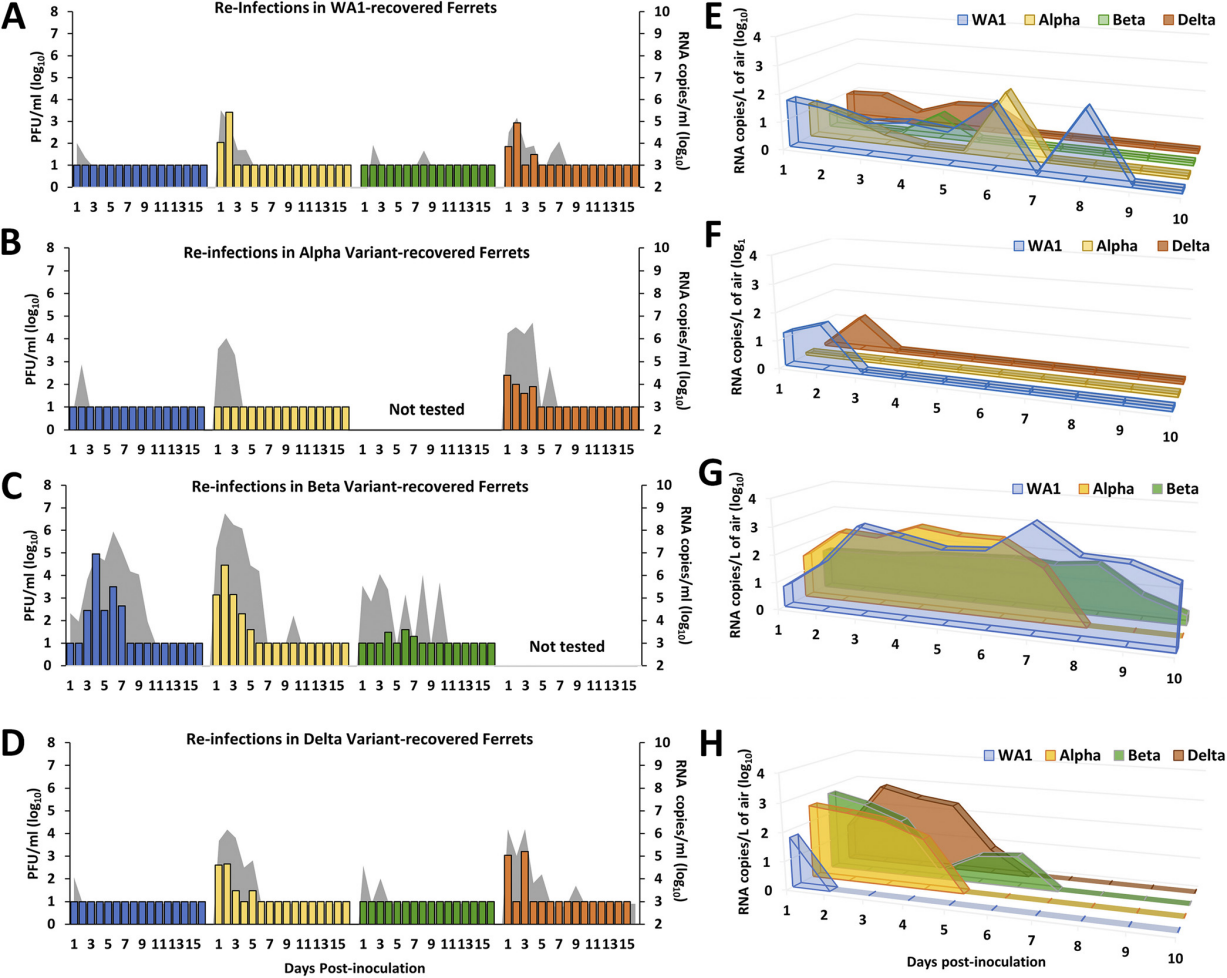
Virus shedding in nasal washes and air samples from ferrets rechallenged with homologous or heterologous SARS-CoV-2. Twelve weeks after primary infection with WA1 (A and E), Alpha (B and F), Beta (C and G), or Delta (D and H) virus, groups of 2 ferrets were rechallenged with 6.0 log_10_ PFU of the indicated SARS-CoV-2 strain (WA1, blue; Alpha, yellow; Beta, green; Delta, orange) for determination of viral titers in NW and exhaled air. Infectious virus in NW was measured using standard plaque assay (left *y* axis, limit of detection = 1.0 log_10_ PFU/mL), and real-time quantitative RT-PCR was used to determine viral RNA load (right *y* axis; gray shading; limit of detection = 2.9 log_10_ RNA copies/mL). Air samples were collected from each inoculated animal daily for 10 days p.i. Following 1 h of air collection using a cyclone sampler, viral RNA was quantified by real-time qRT-PCR, and average titers of positive samples in the >4-μm fraction for each ferret group are shown. Limit of detection was 1 RNA copy/L of air.

Ferrets initially challenged with WA1 virus displayed protection against reinfection with WA1 virus or Beta variant, as infectious virus was not recovered from any NW samples (Fig. 3A). However, infectious virus was recovered from ferrets rechallenged with Alpha or Delta virus, albeit at a level at least a log lower than that measured during the primary infection experiment. Viral RNA was found in NW for all of the rechallenge viruses (3.6 to 5.3 log_10_ copies/mL) and was highest at day 1 p.i., likely in part due to residual inoculum present in upper airways. RS samples were negative for infectious virus but positive for viral RNA only when rechallenged with WA1 or Alpha variant viruses (Table 1). Airborne viral RNA was detected from infected ferrets in all virus groups at peak titers ranging from 0.9 to 2.1 log_10_ copies/L and was detectable for up to 9 days p.i. with homologous WA1 virus (Fig. 3E). ELISAs showed seroconversion to each of the rechallenge viruses at day 28 postreinfection, with normalized IgG levels boosted from prereinfection levels (Table 2).

Ferrets initially challenged with Alpha virus had no detectable infectious virus in NW following rechallenge with either WA1 or Alpha virus. Viral RNA was detected in those samples until day 3 p.i. (<6.0 log_10_ copies/mL), but none was detected from RS samples (Table 1; Fig. 3B). Airborne viral RNA was detected following WA1 (1.4 log_10_ copies/L of air), but not Alpha, virus rechallenge. In contrast, rechallenge with Delta virus resulted in detection of infectious virus and viral RNA in NW (mean peak 2.2 log_10_ PFU/mL and 6.4 log_10_ RNA copies/mL, respectively). Delta viral RNA, but no infectious virus, was detected in the RS of a single ferret, while both animals in this group exhaled viral RNA into air samples (mean peak 1.2 log_10_ copies/L), all of which cleared within a week (Fig. 3F). Seroconversion to WA1, Alpha, and Delta viruses was noted (Table 2). Rechallenge with the Beta variant was excluded because only 6 animals were infected following primary Alpha virus challenge and were allocated to other virus groups.

Homologous rechallenge with Beta variant led to detection of low levels of infectious virus in NW collected between days 3 and 7 p.i. (mean peak 1.5 log_10_ PFU/mL), and viral RNA was detected until day 9 p.i. (mean peak 6.1 log_10_ copies/mL) (Table 1; Fig. 3C). Low levels of Beta virus RNA persisted in the air throughout the 10-day sampling time course (≤1.8 log_10_ copies/L of air). Heterologous challenges of ferrets previously inoculated with Beta variant led to productive infection as evidenced by the detection of infectious WA1 (mean peak 4.7 log_10_ PFU/mL) and Alpha (mean peak 4.2 log_10_ PFU/mL) viruses in NW from all the rechallenged ferrets. Viral RNA was detected in NW and air samples until days 9 and 8 p.i., respectively (Fig. 3G). ELISA revealed seroconversion of all animals postrechallenge, despite no detection of IgG antibodies against the Beta S protein after primary inoculation (Table 2). The Delta virus had yet to emerge at the time of these experiments, and so it was not included as a rechallenge virus for this group.

NW collected from ferrets that were initially challenged with Delta virus and rechallenged with WA1 or Beta virus were mostly negative for infectious virus, and low levels of viral RNA were cleared by day 3 p.i. (mean peak titer ≤4.6 log_10_ copies/mL) (Table 1; Fig. 3D). WA1 virus RNA in the air was detected for only 1 day, whereas Beta virus RNA was detected in air samples until day 6 p.i. (mean peak titers ≤2.8 log_10_ copies/L of air) (Fig. 3H). Initial infection with Delta variant did not protect the ferrets from reinfection with the Alpha virus, as both rechallenged ferrets had infectious virus (2.6 log_10_ PFU/mL) and viral RNA (6.0 log_10_ copies/mL) in NW samples until day 5 p.i. RS were positive for WA1 and Alpha virus RNA, while Alpha virus was detected in the air until day 4 p.i. Homologous challenge with Delta variant also resulted in detectable infectious virus in NW (2.9 log_10_ PFU/mL) from both ferrets as well as viral RNA (6.0 log_10_ copies/mL), and Delta virus shedding into the air was also detected from each of the challenged ferrets (2.8 log_10_ copies/L). Normalized IgG antibodies against the S protein of secondary challenge viruses were detectable prior to rechallenge and, in most cases, were elevated following rechallenge with any of the viruses (Table 2).

With few exceptions, prior exposure to any of the SARS-CoV-2 strains provided some level of protection. Nevertheless, SARS-CoV-2 RNA was detected in airborne particles collected during rechallenge from nearly all the animals (Fig. 3E to H), suggesting that, despite indications of protection, animals were susceptible to reinfection and capable of shedding virus into the environment following rechallenge with homologous or heterologous virus.

### Adaptation of SARS-CoV-2 S protein to ferrets.

Key amino acid substitutions were reported for the SARS-CoV-2 human isolates chosen for this study. Compared with WA1 virus, Alpha, Beta, and Delta lineage viruses acquired the D614G substitution, which has been associated with enhanced receptor binding affinity, infectivity, and transmissibility (12–14). Alpha and Beta variants displayed an N501Y substitution shown to be critical for enhanced receptor binding affinity of the S protein (15, 16), and Delta virus possessed a P681R mutation which confers increased furin cleavage efficiency and cell surface entry (17, 18). To evaluate SARS-CoV-2 adaptation to the ferret host, next-generation sequencing (NGS) technology was employed to characterize virus populations present in samples collected from infected ferrets. Testing was limited to specimens with ≥3 log_10_ viral RNA copies/μL extracted RNA and variant populations detected at >5% frequency; the term variant in this context is not to be confused with a circulating variant virus (e.g., Alpha or Beta). Although this part of the text focuses on major variants of the S protein (detection of >25% frequency of genome copies), full genomes were sequenced, and all variants are listed in Table S3–S6.

10.1128/mbio.02421-22.4 TABLE S3 Major and minor genomic variants detected in WA1 virus-infected ferrets. ^a^The amino acid(s) listed on the left of the residue position substituted for the amino acid(s) listed on the right side. An insertion (ins) or deletion (del) was found at the indicated amino acid position. Spike protein variants at amino acids 1271 to 1274 are not listed. ^b^NT, not tested because viral RNA levels were too low. ND, no variants detected above the 5% threshold cutoff. Download Table S3, PDF file, 0.03 MB. Copyright © 2022 Pulit-Penaloza et al. 2022 Pulit-Penaloza et al. https://creativecommons.org/licenses/by/4.0/ This content is distributed under the terms of the Creative Commons Attribution 4.0 International license.

**FIG 4:**
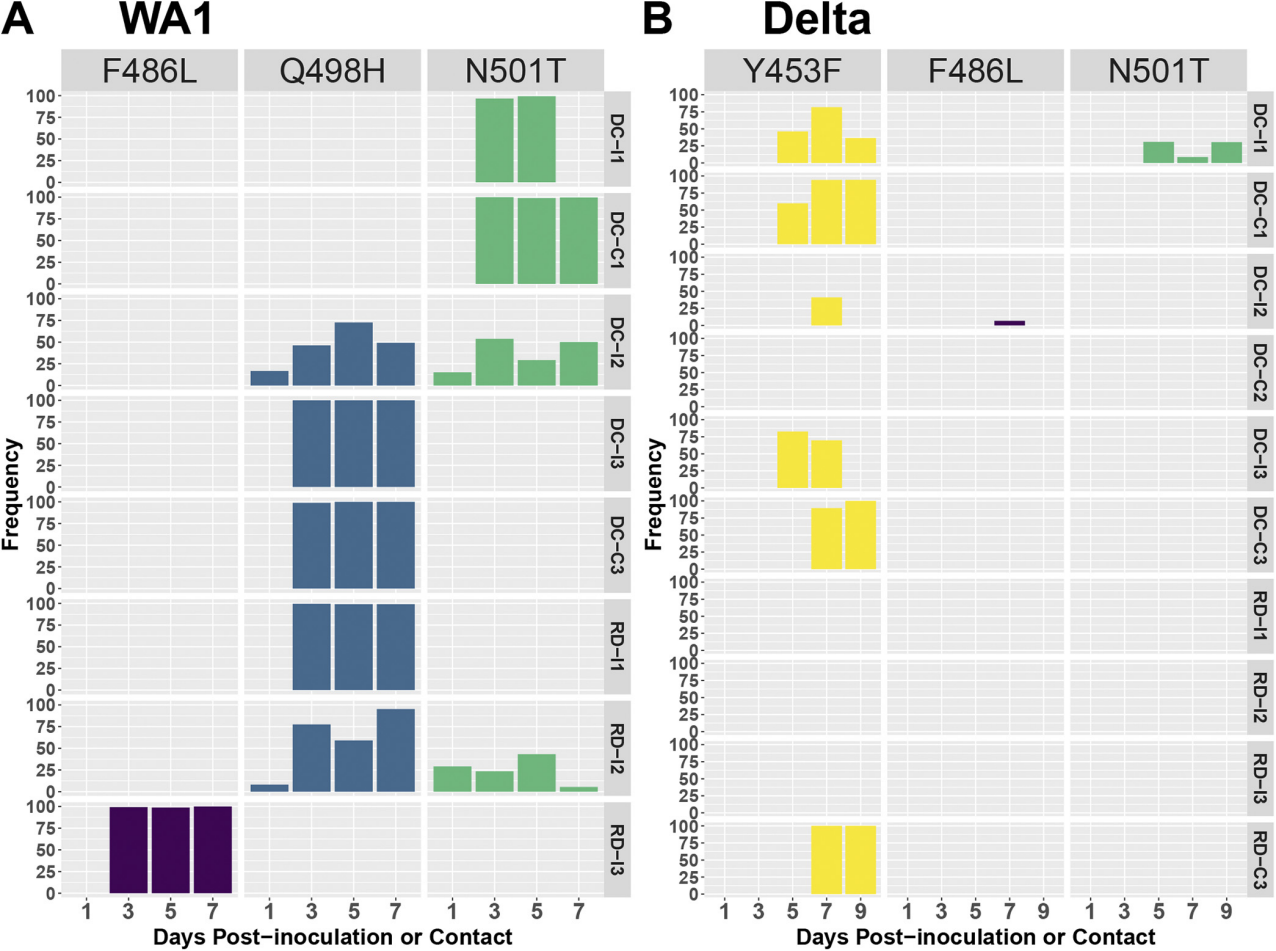
Genomic variants in the RBM of the S protein found in WA1 and Delta virus-infected ferrets during transmission assessments. Viral RNA extracted from nasal washes collected from ferrets inoculated with WA1 (A) and Delta (B) viruses was sequenced by iSeq100 following amplification with the IDT Midnight primer set. Frequencies of genomic variants are shown individually for each ferret, listed on the right side of the panels, and at each time point, shown at the bottom. DC-I, an inoculated animal from the DCT experiment. DC-C, a contact animal from the DCT experiment. RD-I, an inoculated animal from the RDT experiment. RD-C, a contact animal from the RDT experiment. Only those specimens with at least 3.0 log_10_ RNA copies/μL of extracted RNA were analyzed.

Major variants were most often observed in the S protein, especially at the receptor binding motif (RBM; amino acids 438 to 508). Combinations of F486L, Q498H, and N501T major variants were detected in NW samples from all WA1-inoculated ferrets with detection at >75% frequency noted at positions F486L (1/6 ferrets), Q498H (3/6 ferrets), and N501T (1/6 ferrets) starting at day 3 p.i. (Fig. 4A). Interestingly, NW samples from two contact animals that were infected via direct contact with WA1-inoculated ferrets (DC-C1 and DC-C3) bore the same variants (N501T or Q498H) as seen in their respective cage mates with almost 100% frequency as early as day 3 p.c. WA1 virus-inoculated ferrets that were euthanized on day 3 p.i. had variants at one or more of these same amino acid positions in the S protein in NW, nasal turbinate, and soft palate specimens; frequencies ranged from 5.6 to 27.7% (F486L), 48.6 to 98.6% (Q498H), and 7.4 to 37.7% (N501T) (Table S3, WA1). Contact ferret DC-C1 also shed variants with a deletion (674 to 678) in the S protein that appeared with just over 50% frequency late in infection (day 9 p.c.). Ferrets initially challenged with Beta virus and rechallenged with WA1 virus had the Q498H protein variant at >93% frequency in nasal washes collected as early as day 1 p.i. (Table S3, WA1).

With few exceptions, only minor variants with low frequency were detected in Alpha and Beta virus NW specimens from inoculated animals. Exceptions were noted in the RBM of the S protein in the day 5 p.i. NW of an Alpha virus-inoculated DCT donor ferret (F486L at 57.8%) and in NW (Y453F at 69.6%) and nasal turbinate specimens (Q498R at 51.5%) collected from ferrets that were euthanized on day 3 p.i. (Table S4, Alpha). Among ferrets rechallenged with Alpha virus, S protein major variants were detected only in the day 1 p.i. NW of one animal (Rechall-DA-2) that received Delta virus as a primary inoculum (Y498N, D570A, G614D, H681P, and H1118D at 51.3 to 66.4%). Low viral yields in Beta virus primary challenge experiments limited the samples suitable for analysis; reinfection experiments yielded NGS data only in the animals that received Beta virus in both primary and rechallenge settings, and no major variants were detected in the S protein (Table S5).

Among ferrets challenged with Delta virus, variants harboring mutations in the S protein were detected in NW samples from all six inoculated ferrets. Three of these animals shed virus harboring variants in the RBM: Y453F at frequencies ranging from 41.1 to 82.6% (Fig. 4B and Table S6, Delta). Three of the four contact animals that became infected during transmission experiments (DC-C1, DC-C3, and RD-C3) also had Y453F major variants in NW samples at 94.1 to 100% frequency by 7 to 9 days p.c. Taken together, NGS analysis revealed signs of early adaptation of SARS-CoV-2 to the ferret host, especially in the S protein.

## DISCUSSION

Due to the exceptional capacity for sustained transmission among humans displayed by SARS-CoV-2, it is becoming increasingly evident that, like seasonal coronaviruses, variants of SARS-CoV-2 will likely continue to circulate globally (19, 20). Several animal models have been established for studying SARS-CoV-2, notably the golden Syrian hamster model, which has provided critical insight into virus pathogenesis and the dynamics of SARS-CoV-2 transmission. Fewer studies report the characterization of SARS-CoV-2 in the ferret model, but collectively, they agree with our findings and show that ferrets are susceptible to SARS-CoV-2 infection and can be contagious despite exhibiting mild or asymptomatic disease with virus primarily restricted to the upper respiratory tract (5, 21). Sporadic detection of infectious virus, viral RNA, and/or viral antigen in extrapulmonary tissues, including olfactory mucosa and the brain, as well as detection of SARS-CoV-2 viral RNA in intestinal tissue and rectal swabs, has been also reported in the hamster and ferret models (7, 8, 22–24). The limited extrapulmonary spread of infectious virus and generally mild disease in ferrets reported here are supported by the relative lack of lesions in respiratory tract tissues on histopathological examination for all viruses examined; our findings corroborate previous studies in ferrets showing a generally minimal to mild inflammatory response in tissues associated with the highest viral titers and minimal to no change in lower respiratory tract tissues (25–27). Considering that ferrets used in this study were ≤8 months old at the time of first virus inoculation, we cannot rule out age-dependent differences in virus pathogenesis, which warrant further study to fully understand the complexity of the pathogenesis and transmissibility of different SARS-CoV-2 strains (28).

As shown in our DCT model, all contacts cohoused with animals that were inoculated with WA1, Alpha, or Delta virus had detectable viral RNA in at least one NW sample; however, only WA1 virus (2/3) and Delta virus (3/3) contacts had infectious virus in NW and seroconverted to homologous virus. The lack of infectious virus and seroconversion following contact with Alpha virus-inoculated ferrets suggests that the sole detection of viral RNA in cohoused contacts likely represents environmental contamination. Enhanced detection of viral RNA relative to infectious virus in NW specimens is consistent with other studies using this model (8, 29). It is currently not known what the infectious dose requirements are for various SARS-CoV-2 variants and whether this characteristic contributes to differences in transmissibility phenotypes. In the DCT model, contact with contaminated fomites, as well as exposure to virus-laden droplets and droplet nuclei, likely increases the chance of reaching the infectious dose threshold necessary for transmission to a cohoused naive host (29).

Respiratory viruses capable of airborne transmission, notably influenza A viruses, have been detected in the exhaled breath of infected humans (30) and ferrets (31, 32), suggesting that SARS-CoV-2 would share this property. Recently, detection of SARS-CoV-2 in aerosol particles released from experimentally inoculated nonhuman primates and golden Syrian hamsters has been reported (33, 34) and agrees with studies detecting viral material in the exhaled breath of human cases (35). Our study provides further evidence that SARS-CoV-2 can transmit in the absence of direct contact, but unlike other reports, airborne virus was quantified here in tandem with transmissibility assessments. We showed that although all inoculated ferrets released virus-laden aerosols, strain-specific differences were noted in the kinetics and quantity of viral RNA shed into the air. Notably, Delta virus was released into the air at higher viral loads and for longer durations than those of other viruses. This is consistent with the higher initial viral load and longer duration of virus shedding among Delta virus-infected patients than among patients with non-Delta variant infections (36, 37). While WA1-, Alpha-, and Delta-infected ferrets all shed infectious virus in nasal washes with viral RNA detected in the air, only Delta virus was able to transmit via the air to 1/3 contacts. General agreement between NW titers and detection of viral RNA in collected aerosols strongly supports exhaled aerosols as the primary source of airborne particles, though it should be noted that persistence of virus in the environment or ferret fur has been reported previously (22, 29) and could possibly contribute to the levels of viral RNA detected in the air.

The size of airborne virus-laden particles dictates the deposition site, which is an important parameter of virus transmission, especially for pathogens that display tropism for specific cells or sites of the respiratory tract. In general, particles of <5 μm can reach lower respiratory tract surfaces, in contrast to larger particles, which likely are deposited in the upper and central human respiratory mucosa (38, 39). Studies using an aerosol particle sizer showed that naive ferrets or ferrets inoculated with various influenza viruses release particles mainly <1.5 μm in aerodynamic diameter. However, viral RNA was predominantly present in particles in the >4 μm particle fraction. Consistent with this observation, transmission between ferrets was abolished when particles of ≥1.5 μm were captured by an impactor (32). In this study, most of the SARS-CoV-2 viral RNA, regardless of the virus strain, was detected in the first sampler stage, which captured particles in a wide range of sizes (>4 μm), which could potentially facilitate airborne transmission. Small quantities of viral RNA were collected in the <4-μm particle fractions, and these could mediate aerosol transmission, especially considering that SARS-CoV-2 was experimentally shown to have the ability to remain infectious in aerosols for several hours (40). One caveat to these data is that viral RNA is not a direct indicator of the amount of airborne infectious virus. The nature of the collection process by the cyclone aerosol sampler used here, although effective for collection of viral particles, compromises virus viability. With the use of a water-based condensation particle sampler, infectious SARS-CoV-2 was successfully detected in air from a hospital room with COVID-19 patients and from breath of experimentally infected hamsters where average viral RNA levels were shown to be 200-fold higher than those of infectious virus (33, 41). Accurate quantification of infectious virus in aerosols presents a methodological and logistical challenge; nevertheless, measuring airborne viral RNA continues to be an effective gauge of transmission potential, especially in comparative studies.

While most human cases of SARS-CoV-2 from 2020 through 2021 were due to primary infection, continued circulation and emergence of diverse SARS-CoV-2 variants have resulted in increased numbers of both reinfections and breakthrough infections postvaccination (41–43). We found that rechallenge with SARS-CoV-2 generally resulted in reduced detection of infectious virus in ferrets, whereas an overall reduction in severity of disease in humans following reinfection has been reported (44, 45). However, the degree of protection from homologous and heterologous rechallenge, and the magnitude of virus RNA-laden particle levels exhaled by ferrets postrechallenge, was dependent on the primary challenge virus, highlighting the complexity of studying this property as variants continue to emerge. The majority of rechallenge studies conducted in ferret and golden Syrian hamster models to date have evaluated homologous virus reinfection only, or reinfection with only one heterogeneous virus pair (46–49). The findings of our study highlight the need to evaluate multiple challenge viruses during both primary and reinfection scenarios to model human exposure. While our examinations were limited to virus reinfection scenarios, future studies evaluating virus transmission in the context of vaccine breakthrough would also be informative.

NGS analysis revealed signs of early adaptation of SARS-CoV-2 to the ferret host, especially in the S protein. Substantive genomic variants were more often detected in the WA1 and Delta viruses that replicated well and displayed transmission capability in the ferret model, while Alpha and Beta viruses acquired fewer major variants in the RBM of the S protein and were not capable of transmission in this model. Emergence of predominant variant populations during the course of infection suggests that such species may have better fitness or replicative advantages in the ferret host. The S protein major variants detected in samples from ferrets inoculated with WA1 or Delta (Y453F, F486L, Q498H, and N501T) were key residues located at the cellular receptor ACE2 binding interface (50). The S protein variants (Y453F, F486L, and N501T) have previously been reported in studies using experimentally inoculated or naturally infected ferrets or minks (7, 8, 51, 52), and the Q498H variant was identified during virus adaptation experiments in the mouse model (53). Mutations Y453F and N501T have been shown to improve virus utilization of ferret and human ACE2 receptors to enter cells (54, 55), indicating that the difference between ferret and human ACE2 receptors poses a host barrier for SARS-COV-2 infection in ferrets, and adaptative mutations in the S protein RBM are necessary for efficient viral replication in this host. Interestingly, Alpha and Beta variants, sharing an S protein N501Y mutation, which was previously shown to enhance receptor binding to ACE2 receptors (56), especially in combination with the Q498R mutation (reported for the Omicron variant) (57), exhibited reduced ability to replicate in ferrets and rarely gave rise to RBM mutations. It remains undetermined whether being less prone to producing receptor binding variants partially contributed to the lowered viral replication of Alpha and Beta viruses in ferrets compared with that of the WA1 and Delta variants.

Overall, we found the ferret to be a useful model of the assessment of SARS-CoV-2 infection and transmission, revealing enhanced transmissibility of the Delta virus relative to previously detected strains. Although phenotypes displayed in ferrets did not always closely reflect those observed in humans, our comparative analysis of variants revealed clear differences in virus shedding in the air and the potential to evaluate reinfection and/or breakthrough infections using the ferret model. Similar studies using Omicron group viruses would also be beneficial. As different animal models have individual strengths and limitations, investigations of SARS-CoV-2 variants using multiple *in vitro* and *in vivo* models are necessary to fully understand the pathogenesis and transmissibility dynamics of circulating strains, ultimately enabling rapid identification of emerging variants that present a threat to humans. Continued surveillance and risk assessment of emerging SARS-CoV-2 variants will be a critical component for pandemic response and preparedness.

## MATERIALS AND METHODS

### Viruses and cells.

Vero E6 cells expressing serine protease TMPRSS2 were cultured and maintained at 37°C and in a 5% CO_2_ atmosphere (modified protocol from reference 12). Stocks of the original hCoV-19/USA/WA-CDC-02982586-001/2020 (commonly known as USA-WA1/2020, EPI_ISL_404895), hCoV-19/USA/TN-CDC3956481-001/2021 (Alpha virus, EPI_ISL_876595), hCoV-19/USA/WY-CDC-2-3846530/2021 (Beta virus, EPI_ISL_1169500), and hCoV-19/USA/KY-CDC-2-4242084/2021 (Delta virus, EPI_ISL_1823618) were propagated in Vero E6/TMPRSS2 cells as described previously (12) and were exclusivity tested to ensure there was no contamination with influenza viruses.

### Ferret experiments.

Six- to 8-month-old male Fitch ferrets (Triple F Farms, Sayre, PA) were housed in Duo-Flo Bioclean mobile units (Lab Products Incorporated, Seaford, DE) during experimentation. Nine ferrets were inoculated intranasally with 1 mL of 6.0 log_10_ PFU of each of the four viruses diluted in phosphate-buffered saline (PBS). The following day, a serologically naive ferret was placed in the same cage as each of three inoculated ferrets (direct contact transmission model) or in a cage with a perforated side wall adjacent to each of three additional inoculated ferrets (respiratory droplet transmission model) (58). Nasal wash (NW), rectal swab (RS), and conjunctival wash/swab (CW) specimens (59, 60) were collected from the inoculated ferrets every 2 days until day 15 postinoculation (p.i.); NW and RS specimens were collected from contact ferrets every 2 days until day 15 postcontact (p.c.) for virus titer determination. All samples were frozen at −80°C prior to titration. Clinical signs of infection were monitored daily for 21 days p.i. and p.c. Aerosol samples were collected daily for 10 days from each of inoculated ferrets in the respiratory transmission model setup (*n* = 3). The 3 remaining inoculated ferrets in each virus group were euthanized on day 3 p.i. for the assessment of virus spread to pulmonary and extrapulmonary tissues and blood as previously described (61, 62). Convalescent-phase sera were collected from all the ferrets on days 19 to 20 p.i. or p.c.

To evaluate protection against reinfection, 6 inoculated donor ferrets from the transmission studies and up to 2 contact ferrets (contacts with detectable virus in NW samples) were maintained for 12 weeks and rechallenged with 1 mL of 6.0 log_10_ PFU of homologous or heterologous virus diluted in PBS (2 ferrets per group). Following inoculation, the ferrets were observed for clinical signs and symptoms of infection for 15 days; NW and RS samples were collected every second day. Aerosol samples were collected daily for 10 days p.i. from each of the inoculated ferrets. Serum samples were collected the day before and 28 days after rechallenge. All animal procedures were approved by the Institutional Animal Care and Use Committee (IACUC; protocol 3175MAIFERC) of the Centers for Disease Control and Prevention and were conducted in an Association for Assessment and Accreditation of Laboratory Animal Care International-accredited facility.

### Air sample collection procedure.

Each day, prior to other scheduled sample collections, conscious and alert ferrets were individually held in disinfected, enclosed, vented transport containers (23.9 L in size) with a perforated lid for 1 h while air was sampled inside the container at 3.5 L/min (resulting in a 210-L total collection volume). The samplers were attached to the outside of a container holding a single ferret with the sampler inlet protruding 3 to 4 cm into the container. Air samples were collected using a National Institute for Occupational Safety and Health (NIOSH) BC 251 two-stage cyclone aerosol sampler (63) at 3.5 L/min at the same time each day. The first sampler stage collected particles of >4 μm into a disposable 15-mL collection tube (Falcon; 35-2096), the second sampler stage collected particles with a diameter of 1 to 4 μm into a disposable 1.5-mL tube (Fisher Scientific; 02-681-339), and the 37-mm polytetrafluoroethylene filter with 3-μm pores (Millipore; FSLW03700) collected particles with a diameter of <1 μm. Following collection, samplers were disassembled in a class II biological safety cabinet, and viral material was immediately collected in PBS and inactivated in AVL buffer (Qiagen; 19073) according to the manufacturer’s protocol. Samples were stored at −80°C before subsequent RNA extraction and quantification. Samplers were decontaminated with 70% ethanol, rinsed thoroughly with distilled water, and air dried after each sampling day. Each transport container was decontaminated with 70% ethanol after each use.

### Infectious virus quantification.

Infectious virus was quantified using a previously described plaque assay with modifications (64). Briefly, Vero E6/TMPRSS2 cells were seeded 1 day before infection on 12-well plates. When cells were confluent, culture medium was removed, and cells were inoculated with specimens serially diluted in cell infection medium (2% fetal bovine serum [FBS], 100 units/mL penicillin, and 100 μg/mL streptomycin). After 1 h of incubation, inoculum was removed, and cells were overlaid with a 1:1 mix of 2.0% cellulose and 2× Dulbecco’s modified Eagle’s medium (DMEM) supplemented with 10% FBS, 200 units/mL penicillin, and 200 μg/mL streptomycin. After 2 days of incubation at 37°C, cells were fixed with 70% ethanol before staining with crystal violet to observe plaques.

### Viral RNA quantification.

Concurrent with infectious virus titration, 140 μL of each sample was inactivated in 560 μL of AVL buffer (Qiagen; 19073) according to the manufacturer’s protocol and stored at −80°C. Viral RNA was extracted using a QIAamp 96 viral RNA mini-extraction kit (Qiagen) and a QIAcube HT automated high-throughput nucleic acid purification platform (Qiagen) (100-μL elution volume). Each RNA sample (5 μL) was tested in duplicate by real-time reverse transcription-PCR (RT-PCR) using the CDC influenza SARS-CoV-2 multiplex assay, with nucleoprotein (N) as the target gene. To quantify viral copy number in each specimen, a 10-fold serial dilution of synthesized SARS-CoV2 viral RNA (GenBank identifier [ID] MN908947.3) (Twist Bioscience) was included on each plate. Molecular-grade water was used as a negative control. Any specimen with a gene copy number of <1 per μL of extracted RNA was regarded as negative. The mean viral RNA copy was normalized and expressed as RNA copy number/milliliter or gram of specimen.

### Serology.

Enzyme-linked immunosorbent assay (ELISA) was performed as previously described (65) with modifications using OptEIA reagent set B (BD; 550534) and proteins obtained from Sino Biologicals (40589-V08B1 [WA1]; 40589-V08B6 [Alpha]; 40589-V08B11 [Beta]; 40589-V08B16 [Delta]). Briefly, plates were coated with 0.15 μg/mL of SARS-CoV-2 spike S1+S2 ECD-His-tagged recombinant proteins or PBS in a 100-μL volume overnight at 4°C. Next, the plates were washed and blocked for 1 h at 37°C. Serially diluted heat-inactivated sera (56°C for 30 min) were then incubated for 1 h at 37°C. Plates were washed five times, and the secondary antibody goat anti-ferret IgG (pan) H+L(heavy plus light chain)-horseradish peroxidase (HRP) (1 mg/mL; Abcam; ab112770) was added at 100 μL/well at a 1:2,000 dilution and incubated for 1 h at 37°C. Following washing, tetramethylbenzidine (TMB) substrate was added according to the manufacturer’s directions, and the plates were analyzed using Promega GLO Max at an optical density at 450 nm (OD_450_). Positivity cutoffs were determined based on screening of 40 naive prepandemic ferret sera for reactivity to SARS-COV-2 proteins. OD_450_ values for IgG were normalized by subtracting the ELISA cutoff value (negative-control mean OD [0.06 to 0.44] plus 3 times standard deviation [SD]).

### Histopathology.

Necropsy tissues (trachea, lung, brain, liver, spleen, kidney, and gastrointestinal tract) were fixed in 10% neutral buffered formalin, processed for routine paraffin histology, sectioned at 4 μm, and stained by hematoxylin and eosin (H&E). Tissues were evaluated by two veterinary pathologists for inflammatory, degenerative, or other significant histopathologic changes.

### NGS.

Ferret specimens with a viral copy number of >10^3^ per μL of extracted RNA were selected for next-generation sequencing (NGS). In brief, viral RNAs were first reverse transcribed, and viral whole-genome cDNA was then amplified into 29 amplicons of approximately 1.2 kb in length using the SARS-CoV-2 Midnight primer panel (IDT) according to the manufacturer’s protocol. Next, the amplicons were purified with Sera-mag SpeedBeads (Fisher Scientific) followed by NGS library preparation with the Illumina Nextera DNA Flex library preparation kit at half-volume reaction size and sequenced paired-end 150 on iSeq100 (Illumina). The Fastq files obtained from iSeq runs were trimmed using BBDuk and then assembled to SARS-COV-2 reference genomes with Bowtie2 (66) as implemented in Geneious Prime, version 2020.0.5. Variants above 5% frequency with a minimum sequencing depth threshold of 200× coverage were called and exported to Excel for summarization and analysis.

### Data availability.

NGS data are available in the NCBI Sequence Read Archive, BioProject ID PRJNA837611.

10.1128/mbio.02421-22.5 TABLE S4 Major and minor genomic variants detected in Alpha virus-infected ferrets. ^a^The amino acid(s) listed on the left of the residue position substituted for the amino acid(s) listed on the right side. An insertion (ins) or deletion (del) was found at the indicated amino acid position. Spike protein genomic variants at amino acids 1271 to 1274 are not listed. Alpha virus spike protein is numbered based on WA1 virus numbering upon alignment. ^b^NT, not tested because viral RNA levels were too low. ND, no variants detected above the 5% threshold cutoff. Download Table S4, PDF file, 0.03 MB. Copyright © 2022 Pulit-Penaloza et al. 2022 Pulit-Penaloza et al. https://creativecommons.org/licenses/by/4.0/ This content is distributed under the terms of the Creative Commons Attribution 4.0 International license.

10.1128/mbio.02421-22.6 TABLE S5 Major and minor genomic variants detected in Beta virus-infected ferrets. ^a^The amino acid(s) listed on the left of the residue position substituted for the amino acid(s) listed on the right side. An insertion (ins) or deletion (del) was found at the indicated amino acid position. Spike protein genomic variants at amino acids 1271 to 1274 are not listed. Alpha virus spike protein is numbered based on WA1 virus numbering upon alignment. ^b^NT, not tested because viral RNA levels were too low. ND, no variants detected above the 5% threshold cutoff. Download Table S5, PDF file, 0.03 MB. Copyright © 2022 Pulit-Penaloza et al. 2022 Pulit-Penaloza et al. https://creativecommons.org/licenses/by/4.0/ This content is distributed under the terms of the Creative Commons Attribution 4.0 International license.

10.1128/mbio.02421-22.7 TABLE S6 Major and minor genomic variants detected in Delta virus-infected ferrets. ^a^The amino acid(s) listed on the left of the residue position substituted for the amino acid(s) listed on the right side. An insertion (ins) or deletion (del) was found at the indicated amino acid position. Spike protein genomic variants at amino acids 1271 to 1274 are not listed. Alpha virus spike protein is numbered based on WA1 virus numbering upon alignment. ^b^NT, not tested because viral RNA levels were too low. ND, no variants detected above the 5% threshold cutoff. Download Table S6, PDF file, 0.03 MB. Copyright © 2022 Pulit-Penaloza et al. 2022 Pulit-Penaloza et al. https://creativecommons.org/licenses/by/4.0/ This content is distributed under the terms of the Creative Commons Attribution 4.0 International license.
