# Supplementary material for: Comparative Assessment of Severe Acute Respiratory Syndrome Coronavirus 2 Variants in the Ferret Model
Source: mBio. 2022 Sep 22;13(5):e02421-22. doi: 10.1128/mbio.02421-22 (PMC9600705; doi:10.1128/mbio.02421-22)
Supplement: TABLE S1 [file mbio.02421-22-s0005.pdf]

Virus (WHO variant)	Pangolin Lineage	Name in the Study	GISAID ID#	Passage History	Amino Acid Mutations in S Protein
USA-WA1/2020		WA1	EPI_ISL_404895	P5	D614
Alpha	B.1.1.7	Alpha	EPI_ISL_876595	P1	H69/V70del, Y144del, N501Y, A570D, D614G, P681H, T716I, S982A, D1119H
Beta	B.1.351	Beta	EPI_ISL_1169500	P1	D80A, D215G, L241/L242/A243del, K417N, E484K, N501Y, D614G, A701V
Delta	B.1.617.2	Delta	EPI_ISL_1823618	P2-plaque purified	T19R, E156/F157del, R158G, L452R, T478K, D614G, P681R, D950N
