# Supplementary figures and images for: Comparative Assessment of Severe Acute Respiratory Syndrome Coronavirus 2 Variants in the Ferret Model
Source: mBio. 2022 Sep 22;13(5):e02421-22. doi: 10.1128/mbio.02421-22 (PMC9600705; doi:10.1128/mbio.02421-22)

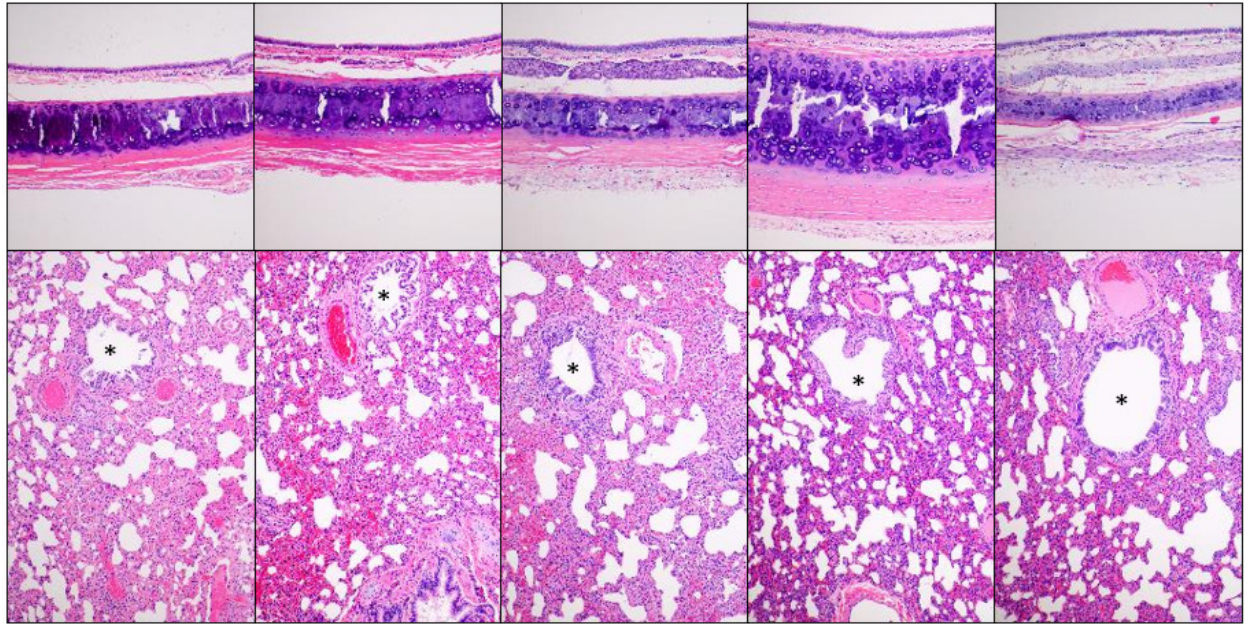

Naïve

WA1

Alpha

Beta

Delta

Supplement: FIG S1 [file mbio.02421-22-s0007.pdf]
