# Supplementary material for: Comparative Assessment of Severe Acute Respiratory Syndrome Coronavirus 2 Variants in the Ferret Model
Source: mBio. 2022 Sep 22;13(5):e02421-22. doi: 10.1128/mbio.02421-22 (PMC9600705; doi:10.1128/mbio.02421-22)
Supplement: TABLE S2 [file mbio.02421-22-s0006.pdf]

Virus	Experiment[b]	Normalized OD_{450} Ferret IgG[a] Inoculated Group (19-20 d.p.i.)[c]	Normalized OD_{450} Ferret IgG[a] Contact Group (19 d.p.c.)[c]
WA1	DCT	1.30	0.68
		0.61	Negative
		0.97	1.04
	RDT	1.14	Negative
		0.90	Negative
		0.84	Negative
Alpha	DCT	2.02	Negative
		1.56	Negative
		2.04	Negative
	RDT	2.09	Negative
		2.03	Negative
		2.09	Negative
Beta	DCT	Negative	Negative
		Negative	Negative
		Negative	Negative
	RDT	Negative	Negative
		Negative	Negative
		Negative	Negative
Delta	DCT	1.70	1.77
		1.50	1.37
		1.70	2.15
	RDT	1.56	Negative
		1.51	Negative
		1.77	1.74
