# Supplementary material for: Comparative Assessment of Severe Acute Respiratory Syndrome Coronavirus 2 Variants in the Ferret Model
Source: mBio. 2022 Sep 22;13(5):e02421-22. doi: 10.1128/mbio.02421-22 (PMC9600705; doi:10.1128/mbio.02421-22)
Supplement: TABLE S3 [file mbio.02421-22-s0001.pdf]

Experiment	Ferret	Amino acid position[a]		Day1-Nasal Wash	Day3-Nasal Wash	Day5-Nasal Wash	Day7-Nasal Wash	Day9-Nasal Wash	Day3-Rectal Swab
				Percentage frequencies of genomic variants[b]					
WA1 virus Transmission Assessment	DC-I1	spike	N501T	NT	96.7	99.3	NT	NT	96.4
		ORF1a	D1554N	NT	ND	9.5	NT	NT	ND
		ORF1b	V1164L	NT	ND	55.5	NT	NT	ND
	DC-C1	spike	N501T	NT	100	99.7	99.7	100	NT
			Y674del	NT	ND	ND	ND	54.8	NT
			Q675del	NT	ND	ND	ND	55.6	NT
			T676del	NT	ND	ND	ND	57.7	NT
			Q677del	NT	ND	ND	ND	56.6	NT
			T678del	NT	ND	ND	ND	52.1	NT
			T678S	NT	ND	ND	ND	5.5	NT
			N679Y	NT	ND	ND	ND	8.1	NT
		ORF1a	V988I	NT	34.9	39.6	8.6	ND	NT
			R3323C	NT	ND	ND	8.4	66.4	NT
	DC-I2	spike	Q498H	16.7	46.2	72.6	49.2	NT	NT
			N501T	15.1	53.8	29.2	50.1	NT	NT
			H655Y	5.6	ND	ND	ND	NT	NT
		ORF1a	T1731I	6.0	ND	ND	ND	NT	NT
		M	T7I	5.5	ND	ND	ND	NT	NT
		ORF8	T11P	18.4	47.1	77.8	53.6	NT	NT
		N	S194T	8.8	ND	ND	ND	NT	NT
	DC-I3	spike	Q498H	NT	100	100	100	NT	NT
			D614N	NT	ND	9.1	ND	NT	NT
		ORF3a	V55F	NT	ND	8.8	ND	NT	NT
		ORF8	T11P	NT	100	99.7	100	NT	NT
	DC-C3	spike	Q498H	NT	98.9	100	100	100	NT
		ORF1a	L3606F	NT	ND	ND	ND	7.4	NT
			A4277T	NT	ND	ND	ND	7.0	NT
		ORF8	T11P	NT	100	100	99.8	100	NT
	RD-I1	spike	Q498H	NT	99.5	99.1	99.1	NT	100
		ORF1a	T224I	NT	99.8	96.9	97.7	NT	ND
			L4386F	NT	7.4	ND	ND	NT	ND
		ORF8	I10T	NT	11	ND	ND	NT	ND
			T11P	NT	99.6	97.7	99.1	NT	99.8
		N	M234V	NT	ND	10.7	9.8	NT	ND
	RD-I2	spike	Q498H	8.1	77.6	59.1	95.3	NT	NT
			N501T	29.1	23.4	43.2	5.4	NT	NT
		ORF1a	V3566I	ND	ND	ND	8.7	NT	NT
		M	T7I	6.0	ND	ND	ND	NT	NT
		ORF8	T11P	17.3	77.4	58.5	95.6	NT	NT
		N	S194T	8.1	ND	ND	ND	NT	NT
			P207T	ND	ND	12.6	41.2	NT	NT
			Q418H	ND	ND	7.4	ND	NT	NT
	RD-I3	spike	F486L	NT	99.3	98.8	100	NT	NT
		ORF1a	V4128A	NT	ND	ND	5.1	NT	NT
		ORF1b	D1130Y	NT	ND	ND	7.0	NT	NT
			I1250del	NT	ND	ND	5.4	NT	NT
			L1531P	NT	ND	ND	9.1	NT	NT
		ORF3a	L94P	NT	ND	ND	6.1	NT	NT
		M	T7I	NT	99.3	98.4	100	NT	NT
		N	S194T	NT	99.1	99.3	99.6	NT	NT
			T393I	NT	99.0	99.6	99.9	NT	NT

Experiment	Ferret	Amino acid position[a]		Day3-Nasal Wash	Day3-Nasal Turbinate	Day3-Soft Palate	Day3-ethmoid
				Percentage frequencies of genomic variants[b]			
WA1 virus Tissue Dissemination Assessment	Nec-1	spike	F486L	ND	12.1	5.6	NT
			Q498H	93.8	76.5	84.6	NT
			N501T	ND	14.2	7.4	NT
			F1256L	19.6	ND	ND	NT
			D1257E	13.2	ND	ND	NT
		ORF1a	L4386F	ND	ND	ND	NT
		M	T7I	ND	ND	8.7	NT
		ORF8	T11P	92.5	80.4	88.7	NT
		N	S194T	ND	8.3	7.5	NT
			T393I	ND	5.7	8.7	NT
	Nec-2	spike	F486L	27.7	ND	9.4	NT
			Q498H	55.7	79.4	48.6	NT
			N501T	18.8	19.6	37.7	NT
			S939F	10.2	13.5	16.5	NT
		M	T7I	29.9	6.7	9.2	NT
		ORF8	T11P	51.8	83.3	52.3	NT
		N	S194T	35.0	ND	7.8	NT
			T393I	29.8	ND	7.1	NT
	Nec-3	spike	Q498H	98.3	98.3	98.6	99.8
		ORF1a	R124C	ND	10.9	11.9	ND
		ORF8	T11P	98.2	97.8	97.8	99.4

Experiment	Ferret	Amino acid position[a]		Day1-Nasal Wash	Day3-Nasal Wash	Day5-Nasal Wash	Day7-Nasal Wash	Day1-Rectal Swab
				Percentage frequencies of genomic variants[b]				
Rechallenge Assessment: Beta virus primary challenge and WA1 virus secondary challenge	Rechall-BW-1	spike	Q498H	NT	99.3	100	98.9	NT
		ORF6	D61V	NT	ND	6.3	ND	NT
			L62ins	NT	ND	5.9	ND	NT
		ORF8	T11P	NT	99.1	100	100	NT
	Rechall-BW-2	spike	V62F	ND	ND	6.1	NT	ND
			HV69L	ND	ND	5.1	NT	ND
			VY143V	ND	ND	6.0	NT	ND
			S221L	ND	5.3	ND	NT	ND
			K304I	ND	ND	7.5	NT	ND
			L452P	ND	ND	ND	NT	6.3
			Q498H	99.9	99.9	93.2	NT	100
			N501Y	ND	ND	7.7	NT	ND
			V551F	ND	ND	5.0	NT	ND
			A570D	ND	ND	7.7	NT	ND
			D614G	ND	ND	6.0	NT	ND
			S640P	ND	ND	7.2	NT	ND
			V642F	ND	ND	5.2	NT	ND
			D1118H	ND	ND	29.6	NT	ND
			N1187I	ND	ND	ND	NT	6.3
		ORF1a	T12I	ND	ND	5.3	NT	ND
			A968P	ND	ND	ND	NT	8.1
			T1001I	ND	ND	11.0	NT	ND
			C1235S	ND	ND	ND	NT	5.6
			F1690V	ND	ND	ND	NT	5.3
			A1708D	ND	ND	5.9	NT	ND
			Q1759H	ND	ND	ND	NT	6.9
			C2160G	ND	ND	ND	NT	9.8
			Y2217F	ND	ND	ND	NT	9.9
			L2218F	ND	ND	ND	NT	6.4
			S2261P	ND	ND	6.5	NT	ND
			Y2281S	ND	ND	ND	NT	8.3
			M2719T	ND	ND	8.2	NT	ND
			R2875L	ND	ND	ND	NT	5.1
			I3369T	ND	ND	8.3	NT	ND
			T3374A	ND	ND	ND	NT	6.4
			D3668Y	ND	ND	ND	NT	5.8
			S3675del	ND	ND	11.5	NT	ND
			G3676del	ND	ND	11.4	NT	ND
			F3677del	ND	ND	11.3	NT	ND
			A3688V	ND	ND	13.3	NT	ND
			R4132G	ND	ND	6.4	NT	ND
			R4179S	ND	ND	6.9	NT	ND
			E4313V	ND	ND	ND	NT	6.3
		ORF1b	L874F	ND	ND	ND	NT	5.4
			Y1505H	ND	ND	ND	NT	10.7
			S1661R	ND	ND	ND	NT	8.5
			C1997S	ND	ND	7.7	NT	ND
		ORF3a	A98G	ND	ND	ND	NT	5.7
		ORF6	M58P	ND	ND	7.5	NT	ND
			D61V	ND	ND	13.0	NT	ND
			L62ins	ND	ND	12.5	NT	ND
		ORF8	T11P	99.2	99.1	93.2	NT	100
			R52I	ND	ND	7.5	NT	ND
			Y73C	ND	ND	6.1	NT	ND
			S84L	ND	ND	7.7	NT	ND
		ORF10	A28V	ND	ND	17.3	NT	7.6
		N	D3L	ND	ND	8.8	NT	ND
			RG203KR	ND	ND	9.8	NT	ND
			S235F	ND	ND	11.1	NT	ND
		M	T7I	ND	ND	ND	NT	5.7
