# Supplementary material for: Comparative Assessment of Severe Acute Respiratory Syndrome Coronavirus 2 Variants in the Ferret Model
Source: mBio. 2022 Sep 22;13(5):e02421-22. doi: 10.1128/mbio.02421-22 (PMC9600705; doi:10.1128/mbio.02421-22)
Supplement: TABLE S4 [file mbio.02421-22-s0002.pdf]

Experiment	Ferret	Amino acid position [a]		D1-nasal wash	D3-nasal wash	D5-nasal wash	D7-nasal wash	D9-nasal wash	D1-rectal swab	D3-rectal swab
				Percentage frequencies of genomic variants [b]						
Alpha virus Transmission Assessment	DC-I1	ORF6	M58L	ND	5.7	NT	NT	NT	ND	NT
			D61V	6.2	7.0	NT	NT	NT	5.7	NT
			D61L	ND	5.7	NT	NT	NT	ND	NT
		ORF1a	T2183I	ND	6.7	NT	NT	NT	6.8	NT
			S2462I	ND	ND	NT	NT	NT	5.1	NT
			A4120T	ND	8.9	NT	NT	NT	ND	NT
		ORF1b	S569L	ND	ND	NT	NT	NT	5.9	NT
			R1729L	ND	ND	NT	NT	NT	6.3	NT
	DC-I2	spike	V367F	ND	ND	9.3	ND	NT	ND	NT
			L390F	ND	ND	5.3	ND	NT	ND	NT
		ORF6	D61V	ND	7.9	21.9	10.1	NT	ND	NT
			L61ins	ND	ND	18	ND	NT	ND	NT
		ORF1a	H1652Y	ND	ND	ND	9.1	NT	ND	NT
			V2133L	ND	ND	ND	7.9	NT	ND	NT
			T2183I	ND	ND	ND	ND	NT	5.5	NT
			C2210F	ND	ND	8.0	ND	NT	ND	NT
			V2238F	ND	ND	ND	5.6	NT	ND	NT
			L2882F	ND	ND	ND	5.7	NT	ND	NT
			V3017F	ND	ND	ND	6.0	NT	ND	NT
			T3586I	ND	ND	7.0	ND	NT	ND	NT
			A3620S	ND	ND	6.7	ND	NT	ND	NT
			A4120T	ND	ND	8.6	ND	NT	ND	NT
		ORF1b	R524L	ND	ND	ND	7.1	NT	ND	NT
			M1499L	ND	ND	11.1	ND	NT	ND	NT
	DC-I3	spike	F486L	ND	ND	57.8	NT	NT	NT	NT
		ORF6	D61V	ND	6.8	8.4	NT	NT	NT	NT
			L62ins	ND	ND	8.3	NT	NT	NT	NT
		ORF1a	G3409C	ND	ND	11.9	NT	NT	NT	NT
			A4120T	ND	6.3	ND	NT	NT	NT	NT
		ORF1b	V551I	ND	ND	5.8	NT	NT	NT	NT
			G1022C	ND	ND	6.3	NT	NT	NT	NT
	RD-I1	spike	S98F	ND	9.8	ND	NT	NT	ND	ND
			A348P	ND	ND	6.7	NT	NT	ND	ND
			I468V	ND	ND	6.2	NT	NT	ND	ND
			D830Y	ND	ND	ND	NT	NT	ND	6.4
			A1080S	ND	ND	ND	NT	NT	ND	6.1
			I1132F	ND	7.5	8.7	NT	NT	ND	ND
			V1230L	ND	ND	ND	NT	NT	ND	ND
			L1234P	ND	ND	ND	NT	NT	ND	5.4
		N	V72F	ND	ND	5.7	NT	NT	ND	ND
		M	I144F	ND	ND	7.3	NT	NT	ND	ND
			D190Y	ND	ND	5.1	NT	NT	ND	ND
			Y204H	ND	ND	5.9	NT	NT	ND	ND
		ORF3a	Q17L	ND	ND	10.8	NT	NT	ND	ND
			G49V	ND	ND	5.1	NT	NT	ND	ND
		ORF6	D61V	5.7	16.2	7.4	NT	NT	ND	9.2
			L62ins	ND	14.9	6.0	NT	NT	ND	8.8
		ORF8	I39T	ND	ND	5.2	NT	NT	ND	ND
		ORF1a	H83del	ND	ND	7.8	NT	NT	ND	ND
			V84del	ND	ND	7.7	NT	NT	ND	ND
			F1353L	ND	ND	8.0	NT	NT	ND	ND
			L1388F	ND	ND	10.3	NT	NT	ND	ND
			T1454I	ND	ND	ND	NT	NT	ND	6.2
			R1645M	ND	5.0	ND	NT	NT	ND	ND
			N1672del	ND	ND	ND	NT	NT	ND	6.6
			E2089D	ND	8.5	ND	NT	NT	ND	ND
			A2128S	ND	13.7	ND	NT	NT	ND	ND
			T2183I	ND	ND	ND	NT	NT	ND	15.5
			D2506E	ND	ND	ND	NT	NT	ND	6.7
			A2916G	ND	ND	ND	NT	NT	ND	5.6
			S2947I	ND	ND	ND	NT	NT	ND	5.0
			N3414del	ND	14.0	ND	NT	NT	ND	ND
			I3415del	ND	14.0	ND	NT	NT	ND	ND
			R3996C	ND	5.2	ND	NT	NT	ND	ND
			A4120T	ND	9.2	11.6	NT	NT	ND	ND
			AQ4145ins	ND	7.0	ND	NT	NT	ND	ND
			R4147V	ND	19.0	ND	NT	NT	ND	ND
			Q4148L	ND	15.8	ND	NT	NT	ND	ND
			C4151Y	ND	8.8	ND	NT	NT	ND	ND
			A4152T	ND	7.2	ND	NT	NT	ND	ND
			P4194T	ND	ND	11.7	NT	NT	ND	ND
			S4196I	ND	5.6	ND	NT	NT	ND	5.9
			F4212V	ND	5.1	ND	NT	NT	ND	ND
			V4485F	ND	ND	5.8	NT	NT	ND	ND
		ORF1b	H353Y	ND	ND	18.1	NT	NT	ND	ND
			K360M	ND	5.0	ND	NT	NT	ND	ND
			A647G	ND	5.6	ND	NT	NT	ND	ND
			Q1204H	ND	ND	9.5	NT	NT	ND	ND
			K1537N	ND	ND	ND	NT	NT	ND	8.2
			D1554E	ND	ND	ND	NT	NT	ND	6.7
			G1568V	ND	ND	ND	NT	NT	ND	6.0
			R1605T	ND	13.7	ND	NT	NT	ND	ND
			M1677T	ND	ND	ND	NT	NT	ND	7.0
			D1973G	ND	ND	27.3	NT	NT	ND	ND
			C2001F	ND	6.5	ND	NT	NT	ND	ND
			N2695I	ND	ND	61.0	NT	NT	ND	ND
	RD-I2	spike	L118F	ND	ND	ND	7.5	NT	NT	NT
			C336S	ND	ND	6.1	ND	NT	NT	NT
			T581K	ND	ND	5.9	ND	NT	NT	NT
			E583D	ND	ND	ND	7.1	NT	NT	NT
			L611P	ND	ND	7.4	ND	NT	NT	NT
			G639V	ND	ND	7.0	ND	NT	NT	NT
			A653V	ND	ND	ND	5.7	NT	NT	NT
			N914D	ND	ND	ND	6.4	NT	NT	NT
			L959F	ND	ND	9.2	ND	NT	NT	NT
			G1204V	ND	ND	ND	8.4	NT	NT	NT
		M	A69G	ND	ND	ND	6.7	NT	NT	NT
		ORF3a	Q57H	ND	ND	5.7	ND	NT	NT	NT
		ORF6	D61V	ND	7.5	6.2	17.1	NT	NT	NT
		ORF1a	H83del	ND	ND	6.1	ND	NT	NT	NT
			V84del	ND	ND	6.1	ND	NT	NT	NT
			L251S	ND	ND	7.9	ND	NT	NT	NT
			L1186P	ND	ND	ND	7.2	NT	NT	NT
			Q1198E	ND	ND	ND	6.4	NT	NT	NT
			Q1224H	ND	ND	ND	7.6	NT	NT	NT
			V1339L	ND	ND	ND	5.8	NT	NT	NT
			S1517F	ND	ND	15.0	ND	NT	NT	NT
			Q1593H	ND	ND	8.7	ND	NT	NT	NT
			I1863V	ND	ND	ND	7.5	NT	NT	NT
			S2488P	ND	ND	ND	5.0	NT	NT	NT
			Y2905H	ND	ND	ND	5.1	NT	NT	NT
			W3600C	ND	ND	8.9	ND	NT	NT	NT
			F3604L	ND	ND	ND	6.3	NT	NT	NT
			F3635V	ND	ND	ND	7.9	NT	NT	NT
			Q3823L	ND	ND	ND	6.2	NT	NT	NT
			V4099L	ND	ND	7.4	ND	NT	NT	NT
			A4120T	ND	ND	8.3	ND	NT	NT	NT
			Y4226C	ND	ND	8.2	ND	NT	NT	NT
			L4295F	ND	ND	ND	18.5	NT	NT	NT
		ORF1b	A167G	ND	ND	ND	5.8	NT	NT	NT
			A366S	ND	ND	ND	5.0	NT	NT	NT
			F497Y	ND	ND	ND	7.0	NT	NT	NT
			A517P	ND	ND	8.3	ND	NT	NT	NT
			V579L	ND	ND	ND	6.3	NT	NT	NT
			H590L	ND	ND	ND	6.7	NT	NT	NT
			V925F	ND	ND	6.6	ND	NT	NT	NT
			M991I	ND	ND	6.4	ND	NT	NT	NT
			V1133F	ND	ND	ND	5.5	NT	NT	NT
			V1144F	ND	ND	10.9	ND	NT	NT	NT
			L1286F	ND	ND	ND	5.2	NT	NT	NT
			A1326S	ND	ND	7.9	ND	NT	NT	NT
			P1342S	ND	ND	ND	6.0	NT	NT	NT
			R1490G	ND	ND	9.0	ND	NT	NT	NT
			T1555I	ND	ND	11.9	ND	NT	NT	NT
			G1626V	ND	ND	7.3	ND	NT	NT	NT
			R1687C	ND	ND	6.7	ND	NT	NT	NT
			T2490M	ND	ND	ND	6.0	NT	NT	NT
	RD-I3	spike	G1246V	ND	ND	ND	NT	NT	28.1	ND
		ORF6	L44V	ND	ND	ND	NT	NT	19.3	ND
			D61V	ND	8.3	ND	NT	NT	18.6	20.9
		ORF7a	E91D	ND	ND	ND	NT	NT	11.4	ND
		N	A155S	ND	ND	ND	NT	NT	ND	7.0
			R185C	ND	ND	ND	NT	NT	6.0	ND
			A398S	ND	ND	ND	NT	NT	ND	10.6
		ORF1a	V23A	ND	ND	ND	NT	NT	ND	13.8
			G989V	ND	ND	ND	NT	NT	20.3	ND
			R1170L	ND	ND	ND	NT	NT	ND	10.1
			H2794L	ND	ND	ND	NT	NT	ND	5.8
			F3557L	ND	ND	16.4	NT	NT	ND	ND
			S3831I	ND	ND	ND	NT	NT	ND	28.1
			A4120T	ND	5.4	9.2	NT	NT	ND	ND
			R4147V	ND	6.5	ND	NT	NT	ND	ND
			F4193S	ND	ND	ND	NT	NT	ND	20.7
		ORF1b	R164L	ND	ND	ND	NT	NT	ND	57.7
			D837H	ND	ND	ND	NT	NT	ND	9.5
			A1190S	ND	ND	ND	NT	NT	ND	16.7
			V1615F	ND	ND	ND	NT	NT	ND	11.8
			H2466D	ND	ND	ND	NT	NT	5.5	ND
			V2481G	ND	ND	ND	NT	NT	ND	6.2

Experiment	Ferret	Amino acid position [a]		Day3-Nasal Wash	Day3-Nasal Turbinates	Day3-Soft Palate	Day3-ethmoid	Day3-Olfactory Bulb	Day3-Brain
				Percentage frequencies of genomic variants [b]					
Alpha virus Tissue Dissemination Assessment	Nec-1	spike	Q498R	10.6	51.5	7.2	ND	NT	NT
		OFR6	M58L	ND	ND	ND	6.0	NT	NT
			D61V	7.1	15.6	9.9	9.2	NT	NT
		ORF1a	A4120T	7.0	ND	ND	10.1	NT	NT
	Nec-2	spike	L981F	ND	ND	ND	5.5	ND	ND
		OFR6	D61V	5.7	ND	5.7	ND	14.6	14.6
		M	F53I	ND	ND	ND	ND	5.7	ND
		ORF1a	T2183I	ND	ND	6.8	ND	15.0	23.4
			A4120T	17.6	6.4	17.6	8.7	ND	ND
	Nec-3	spike	C131W	6.4	ND	ND	ND	ND	NT
			Y453F	69.6	5.2	35.0	ND	ND	NT
			E554D	5.8	ND	ND	ND	ND	NT
			A956S	6.0	ND	ND	ND	ND	NT
			I1081F	ND	ND	ND	ND	6.1	NT
			D1259E	ND	ND	ND	ND	8.4	NT
		OFR6	F22L	ND	ND	ND	ND	5.7	NT
			M58L	ND	ND	5.1	ND	ND	NT
			D61V	10.7	9.1	7.9	11.4	27.0	NT
		ORF3a	S92P	ND	ND	ND	ND	6.0	NT
		ORF1a	L107P	ND	ND	ND	ND	7.5	NT
			G150V	ND	ND	ND	ND	6.6	NT
			K1364N	ND	ND	ND	ND	6.5	NT
			C1711F	ND	ND	ND	ND	6.8	NT
			T1890I	ND	ND	ND	ND	10.8	NT
			S2822P	ND	ND	ND	8.2	ND	NT
			G3458V	ND	ND	ND	ND	6.5	NT
			G3617V	ND	ND	ND	ND	7.9	NT
			A4120T	ND	ND	ND	5.7	23.3	NT
		ORF1b	A397S	ND	ND	ND	ND	6.3	NT
			M2667I	ND	ND	ND	ND	5.3	NT

Experiment	Ferret	Amino acid position [a]		Day1-Nasal Wash	Day3-Nasal Wash	Day5-Nasal Wash	Day7-Nasal Wash	Day9-Nasal Wash	Day1-Rectal Swab	Day3-Rectal Swab	Day5-Rectal Swab
				Percentage frequencies of genomic variants [b]							
Rechallenge Assessment: Delta virus primary challenge and Alpha virus secondary challenge	Rechall-DA-1	spike	L5F	NT	10.8	NT	NT	NT	NT	NT	NT
			F1256L	NT	17.6	NT	NT	NT	NT	NT	NT
		ORF1a	D194Y	NT	17.3	NT	NT	NT	NT	NT	NT
			G1794S	NT	28.5	NT	NT	NT	NT	NT	NT
		ORF1b	L2577P	NT	11.3	NT	NT	NT	NT	NT	NT
		N	D128E	NT	9.1	NT	NT	NT	NT	NT	NT
			K261E	NT	6.3	NT	NT	NT	NT	NT	NT
	Rechall-DA-2	spike	I68IHV	41.3	NT	NT	NT	NT	NT	NT	NT
			V141VY	38.9	NT	NT	NT	NT	NT	NT	NT
			Y498N	65.4	NT	NT	NT	NT	NT	NT	NT
			D570A	64.7	NT	NT	NT	NT	NT	NT	NT
			G614D	66.4	NT	NT	NT	NT	NT	NT	NT
			H681P	51.3	NT	NT	NT	NT	NT	NT	NT
			I716T	34.5	NT	NT	NT	NT	NT	NT	NT
			A982S	35.7	NT	NT	NT	NT	NT	NT	NT
			H1118D	53.3	NT	NT	NT	NT	NT	NT	NT
			F1256L	18.9	NT	NT	NT	NT	NT	NT	NT
		ORF1a	I1001T	61.5	NT	NT	NT	NT	NT	NT	NT
			D1708A	56.9	NT	NT	NT	NT	NT	NT	NT
			T1731I	8.0	NT	NT	NT	NT	NT	NT	NT
			T2230I	54.4	NT	NT	NT	NT	NT	NT	NT
			I4084T	44.3	NT	NT	NT	NT	NT	NT	NT
			A4120T	6.3	NT	NT	NT	NT	NT	NT	NT
		ORF1b	L218P	57.2	NT	NT	NT	NT	NT	NT	NT
			L314P	60.4	NT	NT	NT	NT	NT	NT	NT
		M	T7I	15.3	NT	NT	NT	NT	NT	NT	NT
		ORF6	M58L	13.0	NT	NT	NT	NT	NT	NT	NT
			D61V	17.9	NT	NT	NT	NT	NT	NT	NT
		ORF8	I52R	53.5	NT	NT	NT	NT	NT	NT	NT
			C73Y	52.7	NT	NT	NT	NT	NT	NT	NT
			L84S	51.1	NT	NT	NT	NT	NT	NT	NT
		N	L3D	49.0	NT	NT	NT	NT	NT	NT	NT
			S194T	20.1	NT	NT	NT	NT	NT	NT	NT
			K203R	48.1	NT	NT	NT	NT	NT	NT	NT
			R204G	48.4	NT	NT	NT	NT	NT	NT	NT
			F235S	46.7	NT	NT	NT	NT	NT	NT	NT
Rechallenage Assessment: WA1 virus primary and Alpha virus secondary	Rechall-WA-2	spike	F1256L	18.5	NT	NT	NT	NT	NT	NT	NT
		ORF6	M58L	6.1	NT	NT	NT	NT	NT	NT	NT
			D61V	8.7	NT	NT	NT	NT	NT	NT	NT
			L62ins	7.8	NT	NT	NT	NT	NT	NT	NT
Rechallenge Assessment: Beta virus primary and Alpha virus secondary	Rechall-BA-1	ORF1a	A4120T	8.5	ND	NT	NT	NT	NT	NT	NT
		ORF6	D61V	6.0	5.9	NT	NT	NT	NT	NT	NT
			L62ins	5.3	ND	NT	NT	NT	NT	NT	NT
	Rechall-BA-2	spike	A363S	ND	ND	ND	NT	NT	12.1	ND	ND
			F490L	ND	ND	ND	NT	NT	ND	ND	14.0
			G667V	ND	ND	ND	NT	NT	7.4	ND	ND
			K835R	ND	ND	ND	NT	NT	6.9	ND	ND
			V1268F	ND	ND	ND	NT	NT	ND	ND	15.6
		ORF1a	Y136F	ND	ND	ND	NT	NT	ND	ND	9.3
			N162K	ND	ND	ND	NT	NT	6.0	ND	ND
			R207L	ND	ND	ND	NT	NT	25.6	ND	ND
			Q218H	ND	ND	ND	NT	NT	ND	ND	6.3
			V1019G	ND	ND	ND	NT	NT	ND	ND	19.4
			V1085L	ND	ND	ND	NT	NT	ND	ND	18.4
			Y1183H	ND	ND	ND	NT	NT	ND	8.4	ND
			V1416A	ND	ND	ND	NT	NT	ND	ND	16.5
			I1435F	ND	ND	ND	NT	NT	ND	ND	17.9
			E1826D	ND	ND	ND	NT	NT	11.7	ND	ND
			V2100L	ND	ND	ND	NT	NT	ND	13.3	ND
			T2183I	ND	ND	5.2	NT	NT	ND	11.1	ND
			L2371P	ND	ND	ND	NT	NT	ND	ND	7.4
			Y2391de	ND	ND	ND	NT	NT	ND	6.4	ND
			F2434L	ND	ND	9.2	NT	NT	ND	ND	ND
			F2641de	ND	ND	ND	NT	NT	ND	ND	9.5
			Q2728L	ND	ND	ND	NT	NT	ND	16.1	ND
			P3447S	ND	ND	ND	NT	NT	ND	5.3	ND
			R3480S	ND	ND	ND	NT	NT	ND	6.5	ND
			S3570G	ND	ND	ND	NT	NT	ND	5.9	ND
			L3841F	ND	ND	ND	NT	NT	ND	12.7	ND
			K3863M	ND	ND	ND	NT	NT	ND	ND	13.6
			L3897S	ND	ND	ND	NT	NT	ND	ND	10.9
			F3905L	ND	ND	ND	NT	NT	ND	ND	11.3
			A4013T	ND	ND	ND	NT	NT	ND	ND	9.8
			F4177L	ND	ND	ND	NT	NT	ND	8.7	ND
			C4367F	ND	ND	ND	NT	NT	ND	8.6	ND
			K4462N	ND	ND	ND	NT	NT	ND	ND	13.6
		ORF1b	C554G	ND	ND	ND	NT	NT	ND	5.2	ND
			M558I	ND	ND	6.4	NT	NT	ND	ND	D
			A1225P	ND	ND	ND	NT	NT	12.8	ND	ND
			T1747I	ND	ND	ND	NT	NT	20.9	ND	ND
			V1984M	ND	ND	ND	NT	NT	ND	ND	6.3
			D2522H	ND	ND	ND	NT	NT	ND	ND	5.4
			T2588I	ND	ND	ND	NT	NT	7.3	ND	ND
			W2628L	ND	ND	ND	NT	NT	ND	6.0	ND
			S2658F	ND	ND	ND	NT	NT	ND	ND	8.2
		ORF3a	V97A	ND	ND	ND	NT	NT	ND	10.3	ND
			P240L	ND	ND	ND	NT	NT	ND	ND	8.9
			Q245L	ND	ND	ND	NT	NT	ND	8.7	ND
		ORF6	D30V	ND	ND	ND	NT	NT	ND	ND	6.4
			M58L	ND	ND	6.6	NT	NT	8.0	ND	11.6
			D61V	ND	6.7	11.7	NT	NT	11.6	10.5	20.7
			L62ins	ND	5.9	10.3	NT	NT	11.0	10.2	17.5
		ORF7a	P68A	ND	ND	ND	NT	NT	23.8	ND	ND
		E	N48K	ND	ND	ND	NT	NT	ND	11.4	ND
		N	R385I	ND	ND	ND	NT	NT	ND	ND	5.5
		M	L206S	ND	ND	ND	NT	NT	ND	ND	5.2
