# Supplementary material for: Comparative Assessment of Severe Acute Respiratory Syndrome Coronavirus 2 Variants in the Ferret Model
Source: mBio. 2022 Sep 22;13(5):e02421-22. doi: 10.1128/mbio.02421-22 (PMC9600705; doi:10.1128/mbio.02421-22)
Supplement: TABLE S5 [file mbio.02421-22-s0003.pdf]

Experiment	Ferret	Amino acid position[a]		Percent frequencies of genomic variants[b] Day1-Nasal Wash	Day3-Nasal Wash	Day5-Nasal Wash	Day7-Nasal Wash	Day9-Nasal Wash	Day1-Rectal Swab	Day3-Rectal Swab	Day5-Rectal Swab
Rechallenge Assessment: Beta virus primary challenge and Beta virus secondary challenge	Rechall-BB-1	spike	F92L	NT	7.7	NT	NT	NT	NT	NT	NT
			K462I	NT	6.9	NT	NT	NT	NT	NT	NT
			G832V	NT	6.1	NT	NT	NT	NT	NT	NT
			R847G	NT	6.8	NT	NT	NT	NT	NT	NT
			Q926R	NT	8.2	NT	NT	NT	NT	NT	NT
		ORF1a	H83del	NT	6.9	NT	NT	NT	NT	NT	NT
			V84del	NT	6.7	NT	NT	NT	NT	NT	NT
			G180R	NT	8.5	NT	NT	NT	NT	NT	NT
			W979L	NT	8.6	NT	NT	NT	NT	NT	NT
			Y1135H	NT	8.0	NT	NT	NT	NT	NT	NT
			F2602L	NT	9.5	NT	NT	NT	NT	NT	NT
			V2793L	NT	14.0	NT	NT	NT	NT	NT	NT
			L3086F	NT	12.5	NT	NT	NT	NT	NT	NT
			P3515L	NT	7.4	NT	NT	NT	NT	NT	NT
			M3709I	NT	13.8	NT	NT	NT	NT	NT	NT
			K3840N	NT	8.9	NT	NT	NT	NT	NT	NT
			Y4077H	NT	6.4	NT	NT	NT	NT	NT	NT
			N4398K	NT	6.0	NT	NT	NT	NT	NT	NT
		ORF1b	F685L	NT	5.3	NT	NT	NT	NT	NT	NT
			D842Y	NT	5.5	NT	NT	NT	NT	NT	NT
			V957F	NT	8.0	NT	NT	NT	NT	NT	NT
			C978R	NT	6.1	NT	NT	NT	NT	NT	NT
			G1136C	NT	5.3	NT	NT	NT	NT	NT	NT
			V1375L	NT	7.8	NT	NT	NT	NT	NT	NT
			V2515A	NT	5.5	NT	NT	NT	NT	NT	NT
			D2527Y	NT	5.4	NT	NT	NT	NT	NT	NT
		ORF3a	I20T	NT	26.3	NT	NT	NT	NT	NT	NT
		ORF6	M58L	NT	7.2	NT	NT	NT	NT	NT	NT
			D61V	NT	9.4	NT	NT	NT	NT	NT	NT
			L62ins	NT	8.8	NT	NT	NT	NT	NT	NT
		ORF10	N34D	NT	6.8	NT	NT	NT	NT	NT	NT
		N	D98G	NT	7.5	NT	NT	NT	NT	NT	NT
			W301C	NT	5.5	NT	NT	NT	NT	NT	NT
	Rechall-BB-2	spike	IHV68I	NT	NT	NT	12.5	NT	NT	NT	NT
			A80D	NT	NT	NT	18.4	NT	NT	NT	NT
			G107C	NT	NT	NT	10.2	NT	NT	NT	NT
			VY143V	NT	NT	NT	16.3	NT	NT	NT	NT
			G215D	NT	NT	NT	17.0	NT	NT	NT	NT
			T240TLLA	NT	NT	NT	14.5	NT	NT	NT	NT
			T588A	NT	NT	NT	6.1	NT	NT	NT	NT
			P681H	NT	NT	NT	10.6	NT	NT	NT	NT
			V701A	NT	NT	NT	12.7	NT	NT	NT	NT
			T716I	NT	NT	NT	13.3	NT	NT	NT	NT
			S982A	NT	NT	NT	17.2	NT	NT	NT	NT
		ORF1a	I265T	NT	NT	NT	13.5	NT	NT	NT	NT
			T1001I	NT	NT	NT	14.8	NT	NT	NT	NT
			V1391G	NT	NT	NT	16.8	NT	NT	NT	NT
			A3685V	NT	NT	NT	22.9	NT	NT	NT	NT
			M3709I	NT	NT	NT	83.6	NT	NT	NT	NT
			D3809del	NT	NT	NT	8.7	NT	NT	NT	NT
			Y3810del	NT	NT	NT	8.9	NT	NT	NT	NT
			L3811del	NT	NT	NT	9.0	NT	NT	NT	NT
			V3812del	NT	NT	NT	8.8	NT	NT	NT	NT
			M3908I	NT	NT	NT	17.2	NT	NT	NT	NT
		ORF1b	P218L	NT	NT	NT	19.3	NT	NT	NT	NT
			L242S	NT	NT	NT	8.2	NT	NT	NT	NT
		ORF3a	L171S	NT	NT	NT	16.6	NT	NT	NT	NT
		ORF6	M58L	NT	NT	NT	12.3	NT	NT	NT	NT
			D61V	NT	NT	NT	19.7	NT	NT	NT	NT
			L62ins	NT	NT	NT	18.5	NT	NT	NT	NT
		ORF8	R52I	NT	NT	NT	11.6	NT	NT	NT	NT
			Y73C	NT	NT	NT	12.3	NT	NT	NT	NT
			L121I	NT	NT	NT	15.3	NT	NT	NT	NT
		E	L71P	NT	NT	NT	13.9	NT	NT	NT	NT
		M	S4F	NT	NT	NT	8.2	NT	NT	NT	NT
		N	D3L	NT	NT	NT	14.8	NT	NT	NT	NT
			RG203KR	NT	NT	NT	10.6	NT	NT	NT	NT
			I205T	NT	NT	NT	10.8	NT	NT	NT	NT
			S235F	NT	NT	NT	10.4	NT	NT	NT	NT
