# Supplementary material for: Comparative Assessment of Severe Acute Respiratory Syndrome Coronavirus 2 Variants in the Ferret Model
Source: mBio. 2022 Sep 22;13(5):e02421-22. doi: 10.1128/mbio.02421-22 (PMC9600705; doi:10.1128/mbio.02421-22)
Supplement: TABLE S6 [file mbio.02421-22-s0004.pdf]

				Percent frequency of genomic variants[b]							
Experiment	Ferret	amino acid position[a]		Day1-Nasal Wash	Day3-Nasal Wash	Day5-Nasal Wash	Day7-Nasal Wash	Day9-Nasal Wash	Day11-Nasal Wash	Day13-Nasal Wash	Day15-Nasal Wash
Delta virus Transmission Assessment	DC-I1	spike	T167I	ND	ND	ND	ND	20.0	NT	NT	NT
			G199C	ND	ND	ND	ND	17.3	NT	NT	NT
			G310A	ND	ND	ND	ND	10.3	NT	NT	NT
			Y453F	ND	ND	46.1	81.7	36.5	NT	NT	NT
			N501T	ND	ND	30.9	8.6	30.5	NT	NT	NT
			D1168A	ND	ND	ND	ND	8.6	NT	NT	NT
		ORF1a	A3958V	ND	ND	ND	ND	15.8	NT	NT	NT
			L4033P	ND	ND	ND	ND	10.5	NT	NT	NT
		ORF1b	K254N	ND	ND	ND	ND	7.1	NT	NT	NT
			D795Y	ND	ND	ND	ND	10.0	NT	NT	NT
			G1107C	ND	ND	ND	ND	12.2	NT	NT	NT
			W2587S	ND	ND	ND	ND	12.2	NT	NT	NT
			G2662V	ND	ND	ND	ND	18.4	NT	NT	NT
		ORF3a	E241Q	ND	ND	ND	ND	30.7	NT	NT	NT
		ORF8	P30A	ND	ND	ND	ND	9.4	NT	NT	NT
		N	R88Q	ND	ND	ND	ND	8.1	NT	NT	NT
	DC-C1	spike	Y453F	NT	ND	59.9	94.1	94.3	52.4	NT	NT
			V991L	NT	ND	ND	ND	ND	5.3	NT	NT
		ORF1a	S1306A	NT	ND	6.0	ND	ND	ND	NT	NT
			F3804L	NT	ND	ND	ND	ND	7.6	NT	NT
			A3889T	NT	ND	ND	ND	ND	5.7	NT	NT
		ORF1b	A1428V	NT	ND	ND	ND	ND	5.8	NT	NT
			D1501H	NT	ND	ND	ND	ND	5.5	NT	NT
	DC-I2	spike	Y453F	ND	ND	ND	41.1	NT	NT	NT	NT
			F486L	ND	ND	ND	6.7	NT	NT	NT	NT
		ORF3a	M125I	9.6	ND	5.7	12.0	NT	NT	NT	NT
	DC-C2	spike	V610F	6.4	ND	ND	ND	ND	NT	NT	NT
		ORF1a	S1306A	ND	ND	8.4	6.1	ND	NT	NT	NT
		ORF1b	T76R	8.8	ND	ND	ND	ND	NT	NT	NT
			P1097S	6.1	ND	ND	ND	ND	NT	NT	NT
			D1292Y	7.1	ND	ND	ND	ND	NT	NT	NT
			P2434L	6.6	ND	ND	ND	ND	NT	NT	NT
		M	S4F	ND	ND	ND	7.9	ND	NT	NT	NT
	DC-I3	spike	Y453F	ND	ND	82.6	69.7	ND	NT	NT	NT
		ORF1a	S3G	ND	ND	ND	19.0	ND	NT	NT	NT
		ORF3a	M125I	5.7	5.1	ND	ND	ND	NT	NT	NT
	DC-C3	spike	L5F	NT	ND	ND	ND	ND	6.0	NT	NT
			Y453F	NT	ND	ND	89.5	100	99.4	NT	NT
			V1104L	NT	9.3	ND	ND	ND	ND	NT	NT
		ORF1a	S1306A	NT	ND	ND	ND	6.3	ND	NT	NT
	RD-I1	spike	S172F	ND	ND	ND	ND	7.7	NT	NT	NT
			Y313C	ND	ND	ND	ND	9.3	NT	NT	NT
			P330L	ND	ND	ND	13.8	ND	NT	NT	NT
			V401L	ND	ND	ND	ND	10.9	NT	NT	NT
		ORF1a	R124H	ND	ND	ND	10.0	ND	NT	NT	NT
			M1014T	ND	ND	ND	ND	15.7	NT	NT	NT
			P1284L	ND	ND	ND	ND	15.8	NT	NT	NT
			V3510A	ND	ND	ND	16.1	ND	NT	NT	NT
			K3929E	ND	ND	ND	8.0	ND	NT	NT	NT
			M4009L	ND	ND	ND	8.1	ND	NT	NT	NT
			A4016S	ND	ND	ND	6.7	ND	NT	NT	NT
		ORF1b	Y264N	ND	ND	ND	ND	14.3	NT	NT	NT
			S775L	ND	ND	ND	5.3	ND	NT	NT	NT
			W791C	ND	ND	ND	7.4	ND	NT	NT	NT
			G1014V	ND	ND	ND	6.1	ND	NT	NT	NT
			W2586C	ND	ND	ND	7.5	ND	NT	NT	NT
			R2652K	ND	ND	ND	7.8	ND	NT	NT	NT
			N2695I	ND	ND	ND	ND	64.1	NT	NT	NT
		ORF3a	M125I	ND	ND	ND	13.0	ND	NT	NT	NT
		ORF8	V62L	ND	ND	ND	6.6	ND	NT	NT	NT
		N	V133F	ND	ND	ND	6.0	ND	NT	NT	NT
		ORF10	L16del	ND	ND	ND	ND	6.5	NT	NT	NT
	RD-I2	spike	H207R	ND	ND	ND	ND	11.7	NT	NT	NT
		ORF1a	F3444S	ND	ND	ND	ND	14.2	NT	NT	NT
			E4143D	ND	ND	ND	ND	16.7	NT	NT	NT
		ORF1b	T315I	ND	ND	ND	ND	9.7	NT	NT	NT
			D1036H	ND	ND	ND	ND	6.1	NT	NT	NT
			W1037G	ND	ND	ND	ND	10.7	NT	NT	NT
			N2695I	ND	ND	ND	ND	64.0	NT	NT	NT
		ORF3a	M125I	ND	ND	ND	ND	20.4	NT	NT	NT
	RD-I3	spike	S46L	ND	ND	ND	24.0	NT	NT	NT	NT
			R190M	ND	ND	ND	27.9	NT	NT	NT	NT
			I692T	ND	ND	ND	17.9	NT	NT	NT	NT
			S975G	ND	ND	ND	14.1	NT	NT	NT	NT
			L996F	ND	ND	ND	16.5	NT	NT	NT	NT
		ORF1a	S1306A	ND	6.0	ND	ND	NT	NT	NT	NT
			V1751A	ND	ND	ND	8.8	NT	NT	NT	NT
			Q1784R	ND	ND	ND	10.1	NT	NT	NT	NT
			T1854I	ND	ND	ND	23.0	NT	NT	NT	NT
			D3812Y	ND	ND	ND	10.4	NT	NT	NT	NT
			P4197L	ND	ND	7.4	ND	NT	NT	NT	NT
		ORF1b	V33A	ND	ND	ND	8.7	NT	NT	NT	NT
			K1860R	ND	ND	ND	17.6	NT	NT	NT	NT
		ORF3a	M125I	ND	5.1	ND	ND	NT	NT	NT	NT
			D210G	ND	ND	ND	6.1	NT	NT	NT	NT
		ORF8	F108L	ND	ND	ND	6.7	NT	NT	NT	NT
		N	G63A	ND	ND	ND	ND	NT	NT	NT	NT
	RD-C3	spike	Y453F	NT	NT	NT	100	100	99.5	100	100
			A1025V	NT	NT	NT	ND	ND	ND	ND	12.9
		ORF1a	S1306A	NT	NT	NT	ND	ND	6.1	7.0	5.1
		ORF1b	W281C	NT	NT	NT	ND	ND	ND	ND	7.1
			V329F	NT	NT	NT	ND	ND	ND	ND	7.1
			R1096I	NT	NT	NT	ND	ND	ND	ND	6.8

				Percent frequency of genomic variants[b]									
Experiment	Ferret	amino acid position[a]		Day3-Nasal Wash	Day3-Nasal Turbinates	Day3-Soft Palate	Day3-Ethmoid	Day3-Trachea	Day3-Lung	Day3-Olfactory Bulb	Day3-Brain	Day3-Rectal Swab	Day3-Conjunctiva
Delta virus Tissue Dissemination Assessment	Nec-1	spike	L226S	ND	ND	ND	ND	ND	ND	ND	6.0	ND	NT
			Y453F	13.2	5.2	ND	ND	5.3	6.8	ND	ND	ND	NT
			T573I	ND	ND	ND	ND	ND	ND	5.3	ND	ND	NT
			G1167V	ND	ND	ND	ND	ND	ND	ND	ND	21.8	NT
		ORF1a	S1306A	10.6	7.0	ND	ND	ND	ND	5.9	ND	ND	NT
			C1787G	ND	ND	ND	ND	ND	ND	6.3	ND	ND	NT
			D1865Y	ND	ND	ND	ND	ND	ND	ND	ND	31.5	NT
			G4240S	ND	ND	ND	ND	ND	ND	ND	7.9	ND	NT
		ORF1b	D1735A	ND	ND	ND	ND	ND	ND	ND	12.8	ND	NT
			F1891Y	ND	ND	ND	ND	ND	ND	ND	ND	50.7	NT
			K2579N	ND	ND	ND	ND	ND	ND	ND	ND	7.5	NT
		ORF3a	L52F	ND	ND	ND	ND	ND	ND	ND	10.7	ND	NT
		N	G170C	ND	ND	ND	ND	ND	ND	ND	ND	7.7	NT
			M234I	ND	ND	ND	ND	ND	ND	ND	ND	7.0	NT
	Nec-2	spike	L455S	ND	ND	ND	ND	ND	NT	5.6	ND	NT	ND
			E491K	ND	ND	ND	ND	ND	NT	5.9	ND	NT	ND
			N501T	18.9	ND	ND	ND	6.3	NT	ND	ND	NT	ND
			R577C	ND	ND	ND	ND	ND	NT	ND	5.8	NT	ND
			Y636H	ND	ND	ND	ND	ND	NT	ND	5.9	NT	ND
			L753F	ND	ND	ND	ND	ND	NT	7.4	ND	NT	ND
			I882V	ND	ND	ND	ND	ND	NT	ND	8.0	NT	ND
			I973V	ND	ND	ND	ND	ND	NT	ND	9.1	NT	ND
			E1207V	ND	ND	ND	ND	ND	NT	ND	ND	NT	5.2
			S1261A	ND	ND	ND	ND	ND	NT	7.3	ND	NT	ND
		ORF1a	Q44R	ND	ND	ND	ND	ND	NT	5.0	ND	NT	ND
			H83del	ND	ND	ND	ND	ND	NT	6.7	ND	NT	ND
			V84L	ND	ND	ND	ND	ND	NT	6.6	ND	NT	ND
			Q946L	ND	ND	ND	ND	ND	NT	10.2	ND	NT	ND
			Y947N	ND	ND	ND	ND	ND	NT	11.9	ND	NT	ND
			Q998H	ND	ND	ND	ND	ND	NT	ND	5.7	NT	ND
			G1101C	ND	ND	ND	ND	5.6	NT	ND	ND	NT	ND
			S1306A	5.9	8.5	ND	ND	ND	NT	ND	ND	NT	ND
			S2048P	ND	ND	ND	ND	5.4	NT	ND	ND	NT	ND
			N2054I	ND	ND	ND	ND	ND	NT	6.3	ND	NT	ND
			G3433R	ND	ND	ND	ND	11.0	NT	ND	ND	NT	ND
			L3692P	ND	ND	ND	ND	ND	NT	ND	ND	NT	5.7
			M3695T	ND	ND	ND	ND	ND	NT	ND	5.4	NT	ND
			R3698I	ND	ND	ND	ND	ND	NT	ND	ND	NT	8.5
			D3836Y	ND	ND	ND	ND	5.8	NT	ND	ND	NT	ND
			C4270R	ND	ND	ND	ND	ND	NT	7.9	ND	NT	ND
		ORF1b	V755A	ND	ND	ND	ND	6.2	NT	ND	ND	NT	ND
			D1238V	ND	ND	ND	ND	5.5	NT	ND	ND	NT	ND
		ORF3a	M125I	ND	8.5	ND	ND	5.7	NT	ND	ND	NT	ND
	Nec-3	spike	Q23H	ND	ND	ND	5.0	NT	ND	NT	ND	NT	NT
			T1160R	ND	ND	ND	ND	NT	5.5	NT	ND	NT	NT
		ORF1a	S1306A	7.7	ND	ND	ND	NT	ND	NT	ND	NT	NT
		ORF1b	L2508S	ND	ND	ND	ND	NT	10.8	NT	ND	NT	NT

				Percent frequency of genomic variants[b]							
Experiment	Ferret	amino acid position[a]		Day1-Nasal Wash	Day3-Nasal Wash	Day5-Nasal Wash	Day7-Nasal Wash	Day9-Nasal Wash	Day11-Nasal Wash	Day13-Nasal Wash	Day15-Nasal Wash
Rechallenge Assessment: WA1 virus primary challenge and Delta virus secondary challenge	Rechall-WD-1	spike	F1256L	11.2	NT	NT	NT	NT	NT	NT	NT
		ORF1a	C2288R	5.4	NT	NT	NT	NT	NT	NT	NT
		ORF1b	Q2069H	7.1	NT	NT	NT	NT	NT	NT	NT
			G2071S	7.9	NT	NT	NT	NT	NT	NT	NT
	Rechall-WD-2	spike	G1219C	9.6	NT	NT	NT	NT	NT	NT	NT
			F1256L	19	NT	NT	NT	NT	NT	NT	NT
		ORF1a	G1101C	16.5	NT	NT	NT	NT	NT	NT	NT
			L2234S	7.5	NT	NT	NT	NT	NT	NT	NT
		ORF1b	Q2069H	7.4	NT	NT	NT	NT	NT	NT	NT
Rechallenge Assessment: Alpha virus primary challenge and Delta virus secondary challenge	Rechall-AD-2	spike	G1219C	NT	9.6	NT	NT	NT	NT	NT	NT
			F1256L	NT	19	NT	NT	NT	NT	NT	NT
		ORF1a	G1101C	NT	16.5	NT	NT	NT	NT	NT	NT
			L2234S	NT	7.5	NT	NT	NT	NT	NT	NT
		ORF1b	Q2069H	NT	7.4	NT	NT	NT	NT	NT	NT
Rechallenge Assessment: Delta virus primary challenge and Delta virus secondary challenge	Rechall-DD-1	spike	I794T	39.1	NT	NT	NT	NT	NT	NT	NT
			F1256L	17.8	NT	NT	NT	NT	NT	NT	NT
		ORF1a	C857F	15.4	NT	NT	NT	NT	NT	NT	NT
		ORF3a	M125I	33.1	NT	NT	NT	NT	NT	NT	NT
		M	A85P	22.5	NT	NT	NT	NT	NT	NT	NT
